# Curcumin Equipped Nanozyme‐Like Metal−Organic Framework Platform for the Targeted Atherosclerosis Treatment with Lipid Regulation and Enhanced Magnetic Resonance Imaging Capability

**DOI:** 10.1002/advs.202309062

**Published:** 2024-05-02

**Authors:** Fanzhen Lv, Huaqiang Fang, Li Huang, Qingqing Wang, Shuangyuan Cao, Wenpeng Zhao, Zhibin Zhou, Weimin Zhou, Xiaolei Wang

**Affiliations:** ^1^ Department of Vascular Surgery the Second Affiliated Hospital Jiangxi Medical College Nanchang University Nanchang Jiangxi 330006 China; ^2^ School of Pharmacy Nanchang University Nanchang Jiangxi 330006 China; ^3^ The National Engineering Research Center for Bioengineering Drugs and the Technologies Institute of Translational Medicine Nanchang University Nanchang Jiangxi 330006 China

**Keywords:** atherosclerosis, magnetic resonance imaging, metal−organic frameworks, reactive oxygen species scavenging, targeted drug delivery

## Abstract

Atherosclerotic cardiovascular disease (ASCVD) has become the leading cause of death worldwide, and early diagnosis and treatment of atherosclerosis (AS) are crucial for reducing the occurrence of acute cardiovascular events. However, early diagnosis of AS is challenging, and oral anti‐AS drugs suffer from limitations like imprecise targeting and low bioavailability. To overcome the aforementioned shortcomings, Cur/MOF@DS is developed, a nanoplatform integrating diagnosis and treatment by loading curcumin (Cur) into metal−organic frameworks with nanozymes and magnetic resonance imaging (MRI) properties. In addition, the surface‐modification of dextran sulfate (DS) enables PCN‐222(Mn) effectively target scavenger receptor class A in macrophages or foam cells within the plaque region. This nanoplatform employs mechanisms that effectively scavenge excessive reactive oxygen species in the plaque microenvironment, promote macrophage autophagy and regulate macrophage polarization to realize lipid regulation. In vivo and in vitro experiments confirm that this nanoplatform has outstanding MRI performance and anti‐AS effects, which may provide a new option for early diagnosis and treatment of AS.

## Graphical abstract

A nanoplatform based on metal−organic frameworks is developed for magnetic resonance imaging diagnosis and treatment of atherosclerotic cardiovascular disease, which functions by scavenging excessive reactive oxygen species in the local plaque microenvironment, inhibiting macrophage apoptosis induced by oxidative stress, promoting macrophage autophagy, and regulating macrophage polarization to effectively reduce lipid influx and enhance efflux.

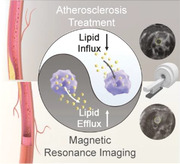

## 1 Introduction

Atherosclerotic cardiovascular disease (ASCVD) is a prevalent and serious health concern that has become the primary cause of mortality and morbidity in humans.^[^ ^1^ ^]^ ASCVD, which usually presents asymptomatically, can be challenging to diagnose in the early stages. Plaque formation is the central pathological process of ASCVD.^[^ ^2^ ^]^ As the progression and burden of plaques escalates, the likelihood of vascular stenosis or vulnerable plaque formation rises, ultimately heightening the risk of acute cardiovascular events.^[^ ^3^ ^]^ Therefore, prompt diagnosis and proper management of atherosclerosis (AS) are imperative to reduce the incidence of ASCVD.

AS is a chronic inflammatory condition in which macrophages play a key role through lipid uptake and accumulation, the production of inflammatory mediators, autophagy and polarization.^[^ ^4^ ^]^ In the early stages of AS, low‐density lipoprotein (LDL) is oxidized by excess reactive oxygen species (ROS) in the arterial intima, resulting in the formation of oxidized LDL (ox‐LDL), which serves as the primary initiating factor in the pathological progression of AS.^[^ ^5^ ^]^ Subsequently, macrophage phagocytosis of ox‐LDL mediated by the scavenger receptor (SR) results in the formation of foam cells (FCs), marking the initiation of AS.^[^ ^6^ ^]^ In addition, macrophage autophagy play a critical role in the regulation of AS progression by promoting the transport of lipid droplets to lysosomes for degradation and efflux of free cholesterol from FCs.^[^ ^7^ ^]^ Similarly, macrophage phenotypes are closely linked to AS, and transforming M1 macrophages to M2 macrophages can have a positive impact on AS management.^[^ ^8^ ^]^ Therefore, simultaneously scavenging ROS to reduce ox‐LDL formation, inhibiting the inflammatory response, facilitating M2 polarization of macrophages and promoting macrophage autophagy are promising treatment strategies for effectively hampering the progression of AS.

Dyslipidemia has been identified as a significant risk factor for the occurrence and progression of ASCVD.^[^ ^9^ ^]^ Research has demonstrated that even when patients achieve target levels of LDL cholesterol through statin therapy, up to 40% still encounter a notable risk of life‐threatening cardiovascular events, known as “residual risk”.^[^ ^10^ ^]^ Curcumin (Cur), a key component in traditional medicine in Asia, is also known as “Indian Saffron” or “Gold Spice” due to its vibrant yellow.^[^ ^11^ ^]^ Research has revealed its potential in inhibiting inflammation, scavenging free radicals, and reducing blood lipids.^[^ ^12^ ^]^ These characteristics make it a potentially valuable alternative to statins, which primarily focus on lowering LDL levels. However, its clinical application is constrained by issues such as low absorption and bioavailability. Encouragingly, recent advancements in nanomaterials have provided new treatment approaches to overcome these challenges.^[^ ^13^ ^]^ In particular, metal−organic frameworks, which possess intramolecular pores, have been found to effectively load target drugs.^[^ ^14^ ^]^ Among them, PCN‐222(Mn) (MOF) exhibits noteworthy superoxide dismutase^[^ ^15^ ^]^ and peroxidase^[^ ^16^ ^]^ activities, as well as an exceptionally high relaxivity,^[^ ^17^ ^]^ making it a promising candidate for AS treatment and plaque magnetic resonance imaging (MRI). Dextran sulfate (DS) is a biocompatible anionic dextran compound extensively utilized for nanoparticle modification.^[^ ^18^ ^]^ It serves as a ligand for SR class A (SR‐A), which is overexpressed in AS plaques, showcasing promising applications in AS therapy.^[^ ^19^ ^]^

**Scheme 1:**
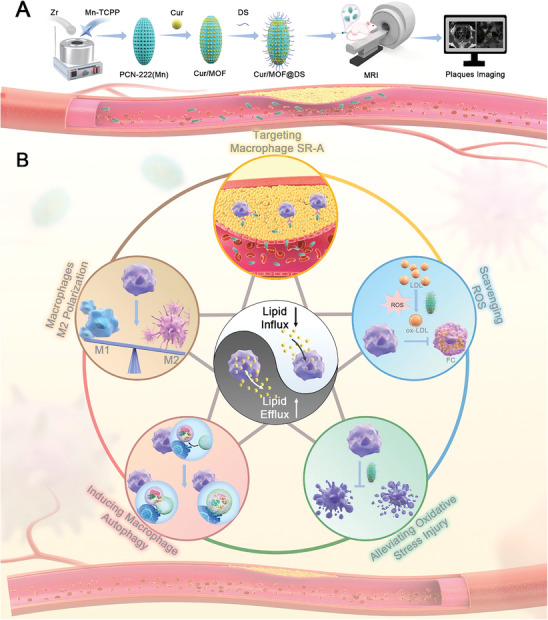
Schematic illustration of Cur/MOF@DS for treatment and enhanced MRI of AS. A) The synthetic process and MRI performance for Cur/MOF@DS, involving the sequential steps of MOF synthesis, Cur loading, surface modification with DS, and in vivo MRI. B) Potential mechanism of Cur/MOF@DS in the treatment of AS: targeted delivery to macrophages or FCs at the plaque region via SR‐A; scavenging excessive ROS in the local plaque microenvironment; inhibiting macrophage apoptosis induced by oxidative stress; promoting macrophage autophagy; modulating macrophage polarization, reducing lipid influx and enhancing efflux.

In this study, we developed Cur/MOF@DS, a nanoplatform designed for the targeted delivery of treatment agents to AS plaque and equipped with MRI capabilities (**Scheme** **1**A). MOF, a nanoparticle characterized by both nanozyme and MRI capabilities, was specifically designed to deliver the anti‐AS drug Cur. Furthermore, MOF was surface‐modified with DS to enhance targeted delivery to macrophages or FCs at the plaque region via SR‐A.^[^ ^20^ ^]^ Both in vitro and in vivo experiments demonstrated that Cur/MOF@DS not only exhibited excellent MRI performance, but also effectively treated AS by scavenging excessive ROS in the local plaque microenvironment, inhibiting macrophage apoptosis induced by oxidative stress, promoting macrophage autophagy and modulating macrophage polarization to reduce lipid influx and enhance efflux (Scheme 1B). To the best of our knowledge, this is the first application of MOF combined with traditional Chinese medicine monomers for AS treatment.

## 2 Results and Discussion

### 2.1 Preparation and Characterization of Cur/MOF@DS

**Figure 1:**
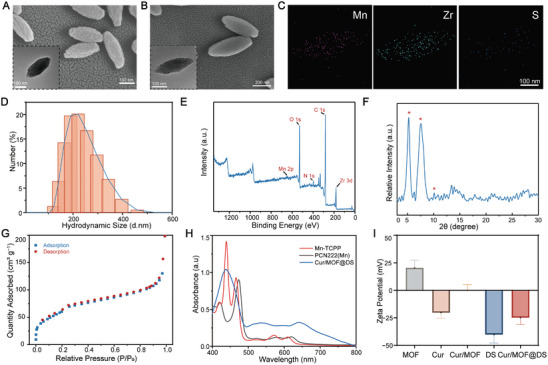
Characterization of Cur/MOF@DS. A,B) SEM and TEM (inset) images of MOF and Cur/MOF@DS. C) Elemental mapping images of Cur/MOF@DS. D) Hydrodynamic diameter distribution curves of MOF. E) XPS spectral of MOF. F) X‐ray diffraction (XRD) patterns of MOF. G) Nitrogen adsorption and desorption isotherms at 77 K of MOF. H) UV–vis spectra of Mn‐TCPP, MOF, and Cur/MOF@DS in ethanol. I) Zeta potentials of MOF, Cur, Cur/MOF, DS, and Cur/MOF@DS in water. Data were presented as means ± standard deviation (SD) (*n* = 3).

MOF was synthesized using a hydrothermal method.^[^ ^21^ ^]^ Scanning electron microscopy (SEM) and transmission electron microscopy (TEM) images revealed that MOF had an ellipsoidal shape with dimensions of ≈240 nm × 90 nm (**Figure** **1**A). Energy‐dispersive X‐ray spectroscopy (EDS) (Figure S2, Supporting Information) confirmed the distribution of C, Mn, Zr, and O in MOF. Meanwhile, the SEM and TEM result revealed that the morphology of Cur/MOF@DS remained unchanged after Cur loading and DS surface modification (Figure 1B). However, the element mapping results confirmed the presence of sulfur, suggesting that DS was modified on the surface of MOF (Figure 1C). Dynamic light scattering measurements indicated that the particle size distribution curve was consistent with TEM results (Figure 1D). X‐ray photoelectron spectroscopy (XPS) analysis further verified the presence of Mn in MOF (Figure 1E). X‐ray diffraction pattern (XRD) displayed diffraction peaks consistent with reported literature (Figure 1F).^[^ ^16^ ^]^ Brunauer Emmett Teller analysis demonstrated a type‐II adsorption isotherm for MOF (Figure 1G) and Cur/MOF@DS (Figure S3, Supporting Information). In the ultraviolet‐visible (UV−vis) spectrophotometry (Figure 1H), MOF exhibited three slightly red‐shifted Q bands, indicating the coordination of Mn ion within the porphyrin molecules.^[^ ^22^ ^]^ It was noted that the zeta potential of Cur/MOF@DS was lower than that of Cur/MOF, confirming the successful modification of Cur/MOF with DS (Figure 1I). Next, in the Fourier‐transform infrared (FTIR) spectrum of Cur/MOF@DS (Figure S4, Supporting Information), the presence of the stretching vibration peak of the carbonyl group C═O at 1699 cm⁻¹ and the vibration peak of the aromatic ring C═C bond at 1595 cm⁻¹ indicated the successful loading of Cur into MOF. Furthermore, the vibrational peak at 1540 cm⁻¹, associated with the ester group and carboxylic acid functional groups, which were characteristic features of DS, confirmed the successful modification of MOF with DS. These results collectively demonstrated the successful construction of the nanoplatform. Subsequently, we assessed the drug loading capacity of the MOF for Cur through UV−vis spectroscopy, revealing an approximate loading capacity of 16.69% (Figure S5, Supporting Information). Finally, in vitro drug release experiments illustrated the release profile of Cur (Figure S6, Supporting Information), and the results revealed that the nanoplatform exhibits a sustained‐release effect for the drug.

### 2.2 ROS Scavenging and MRI Performance of MOF and Cur/MOF@DS

**Figure 2:**
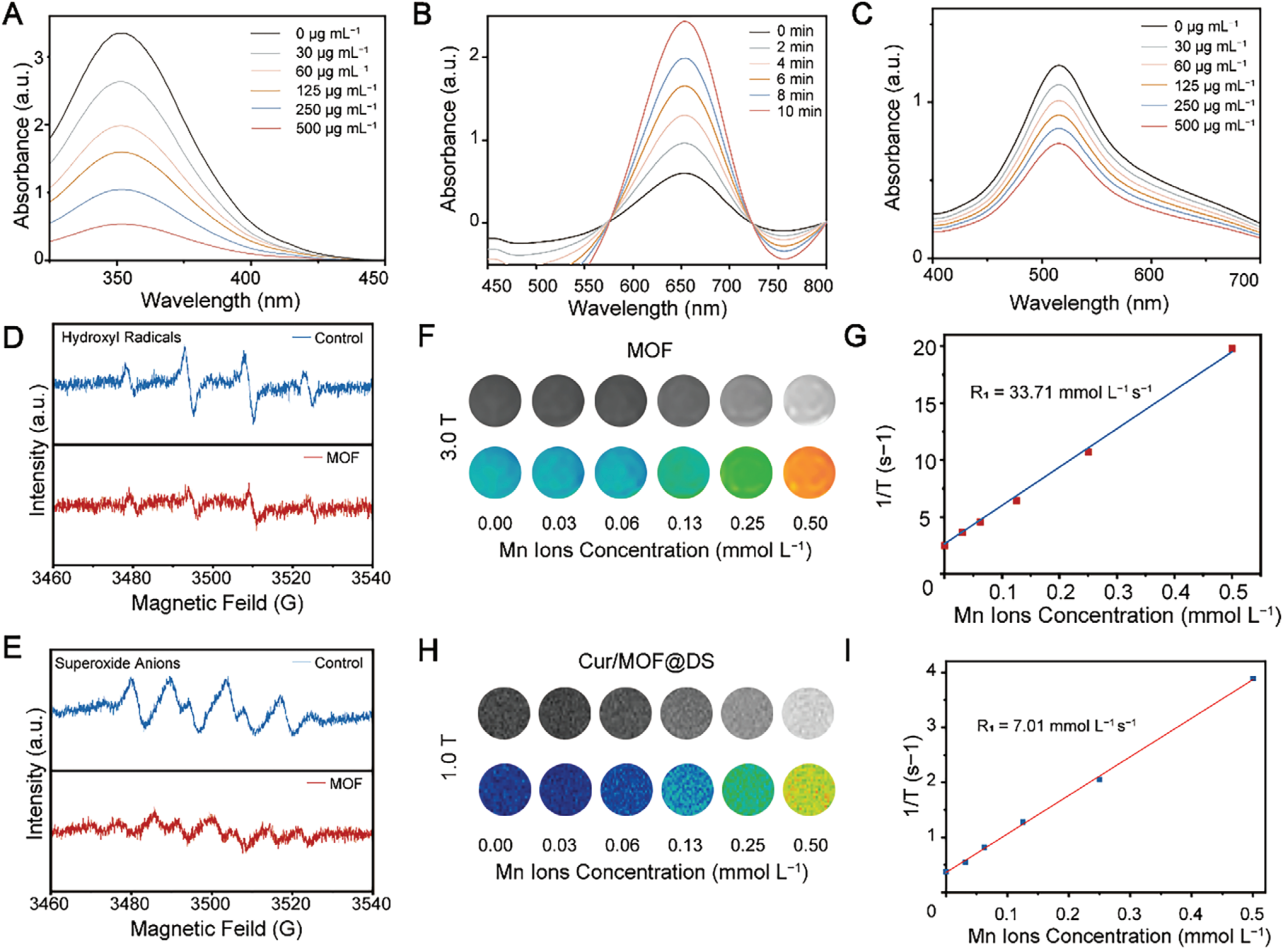
ROS scavenging and MRI properties of MOF and Cur/MOF@DS. A) Evaluation of hydrogen peroxide scavenging ability at varying Cur/MOF@DS concentrations in 1 mm H_2_O_2_ and 1 m potassium iodide (KI) solution. B) Evaluation of peroxide scavenging ability after different reaction time by mixed Cur/MOF@DS and 0.1 mm TMB with 1 mm H_2_O_2_ solution. C) Evaluation of DPPH radical scavenging ability at different Cur/MOF@DS concentrations in 1 mm DDPH free radical scavenger solution. D,E) ESR spectroscopy detection of the scavenging ability of MOF for hydroxyl radicals and superoxide anions of the mixed solution of 5,5‐dimethyl‐1‐pyrroline‐N‐oxide (DMPO), titanium dioxide (TiO_2_) and MOF solutions. F) MRI of MOF with varying concentrations of Mn ions using a 3.0 T MR scanner. G) Functional relationship between the Mn concentration and 1/T of MOF (3.0 T). H) MRI of Cur/MOF@DS with varying concentrations of Mn ions concentration using a 1.0 T MR scanner. I) Functional relationship between the Mn ions concentration and 1/T of MOF (3.0 T).

The in vitro ROS scavenging potential of MOF and Cur/MOF@DS were evaluated. UV−vis spectroscopy was employed to assess the hydrogen peroxide (H_2_O_2_) degradation capacity of MOF and Cur/MOF@DS at various concentrations. The results revealed that MOF and Cur/MOF@DS displayed noteworthy catalase activity, and a positive correlation was observed between the degradation activity and concentration of MOF (Figure S7A, Supporting Information) and Cur/MOF@DS (**Figure** **2**A). Similarly, MOF (Figure S7B, Supporting Information) and Cur/MOF@DS (Figure 2B) demonstrated notable peroxidase activity, and the generation of oxidized 3,3′,5,5′‐tetramethylbenzidine (TMB) was positively correlated with the reaction time. Additionally, the 2‐diphenyl‐1‐picrylhydrazyl (DPPH) radical scavenging assay revealed that MOF (Figure S7C, Supporting Information) and Cur/MOF@DS (Figure 2C) were effective in scavenging free radicals, and the ability was enhanced with increasing MOF and Cur/MOF@DS concentrations. The aforementioned results showed that Cur/MOF@DS exhibits enhanced ROS scavenging capability compared to MOF alone, suggesting a synergistic effect between Cur and MOF in promoting ROS scavenging. Subsequently, electron spin resonance (ESR) analyses were conducted to evaluate the ability of MOF to eliminate hydroxyl radicals and superoxide anions. These results showed a substantial decrease in the ESR signal intensity following MOF treatment, affirming its ability to effectively eliminate hydroxyl radicals (Figure 2D) and superoxide anions (Figure 2E).

Given the paramagnetic properties of manganese (Mn), a study was conducted to evaluate the MRI performance of MOF to explore its potential application in the early diagnosis of AS plaque. The concentration of Mn ion in MOF and Cur/MOF@DS were determined using inductively coupled plasma optical emission spectrometer (ICP‐OES) analysis. Subsequently, T1‐weighted images of MOF at various Mn ion concentrations (0.00, 0.03, 0.06, 0.13, 0.25 and 0.50 mmol L^−1^) were obtained using a 3.0 Tesla (T) magnetic resonance (MR) scanner. The MRI results demonstrated a correspondence between T_1_‐weighted MR signal and MOF concentration (Figure 2F). The longitudinal relaxation rate (R1), which can reflect the MRI effect of T1‐weighted images, was calculated to be 33.71 mmol L^−1^ s^−1^ (Figure 2G). Furthermore, to assess the MRI capability of Cur/MOF@DS, we additionally measured the T1 relaxation time and obtained T1‐weighted images on a 1.0 T MR scanner. The results illustrated that, even after Cur loading and DS surface modification, the T1‐weighted MR signal of Cur/MOF@DS remains concentration‐dependent (Figure 2H), with a R1 value of 7.01 mmol L^−1^ s^−1^ (Figure 2I). These findings suggest that Cur/MOF@DS not only exhibits outstanding in vitro ROS scavenging activity but also possesses noteworthy MRI capabilities. This underscores its potential application in the treatment of inflammatory diseases.

### 2.3 In Vitro Macrophage Targeting and Anti‐Inflammatory Effects of Cur/MOF@DS

**Figure 3:**
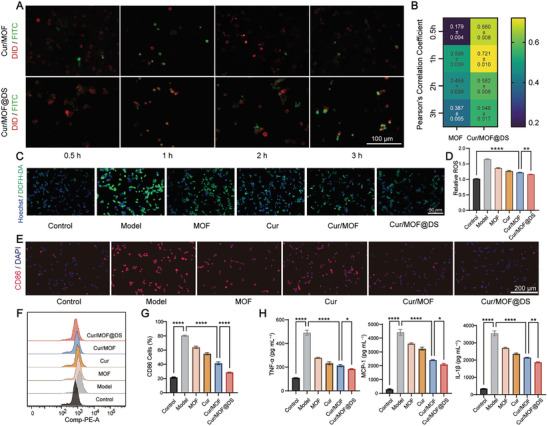
In vitro targeting macrophage and anti‐inflammatory effects of Cur/MOF@DS. A) Fluorescence colocalization assay to examine the phagocytosis of Cur/MOF and Cur/MOF@DS by macrophages. Red fluorescence represented DID‐stained cell membranes, and green fluorescence represented FITC‐labeled nanoparticles. B) Quantitative analysis using pearson's correlation coefficient to measure co‐localization relationship between FITC and DID. C) ROS fluorescence assays of Raw264.7 cells in different treatment groups. Blue fluorescence represented Hoechst‐stained nuclei, and green fluorescence represented ROS stained by DCFH‐DA. D) Quantitative analysis of the relative content of ROS in different treatment groups. E) CD86 fluorescence staining of Raw264.7 cells after different treatments. Red fluorescence is the Cyanine 3 (Cy3)‐labeled secondary antibody and blue fluorescence is the nucleus after DAPI staining. F,G) Quantification of CD86 fluorescence intensity of Raw264.7 cells by propidium iodide (PI) flow cytometric assay. H) Concentrations of three inflammatory factors in Raw264.7 cells supernatants after different treatments. Data were presented as means ± SD (*n* = 3). *P < 0.05, **P < 0.01, ****P < 0.0001.

It has been observed that during the progression of AS, SR‐A is overexpressed on activated macrophages instead of quiescent macrophages or intrinsic vascular cells.^[^ ^23^ ^]^ Additionally, DS, a negatively charged macromolecule, has been identified as a specific ligand for SR‐A through electrostatic interactions.^[^ ^24^ ^]^ In previous work,^[^ ^25^ ^]^ we have verified that DS‐modified nanoparticles bind selectively to SR‐A receptors by conducting competitive inhibition experiments, where free DS and DS‐modified materials were competitively bound to the activated SR‐A receptors on the surface of Raw 264.7 cells. Therefore, DS were surface‐modified on nanoparticles to target SR‐A on the surface of macrophages or FCs within the plaque region. The surface binding of SR‐A to Cur/MOF@DS was investigated via fluorescence colocalization. Fluorescein isothiocyanate (FITC) pre‐labeled Cur/MOF and Cur/MOF@DS were co‐cultured with mouse mononuclear macrophages cells (Raw264.7 cells) activated by lipopolysaccharide (LPS) respectively. Subsequently, cell membrane far‐infrared fluorescent probes (1,1′‐Dioctadecyl‐3,3,3′,3′‐tetramethylindodicarbocyanine perchlorate, DID) were employed for cell membrane labeling. The results showed that the phagocytosis efficiency of the nanoplatform by macrophages was highest in the Cur/MOF@DS group, with a higher Pearson's correlation coefficient. This finding confirmed a significant increase in the phagocytosis efficiency of Raw264.7 cells against the nanoplatform after surface modification with DS (**Figure** **3**A,B).

Moderate levels of ROS serve critical physiological functions in organisms and participate in various physiological processes, including cell signaling and immune defenses.^[^ ^26^ ^]^ However, excessive ROS levels can cause oxidative damage to the cells and tissues. To evaluate the ROS scavenging ability of Cur/MOF@DS in Raw264.7 cells, an in vitro cellular inflammation model was established by stimulating the cells with LPS. ROS generation was monitored using 2′,7′‐dichlorofluorescein diacetate (DCFH‐DA), and the cell nuclei were labeled with Hoechst 33 342 staining solution. After the co‐incubation of LPS and Raw264.7 cells for 4 h, the Model group displayed a significant increase in ROS levels. In contrast, a notable decrease in the green fluorescence signal was observed among the groups preincubated with different materials, with the Cur/MOF@DS group showing the most significant effect (Figure 3C,D; Figure S8A, Supporting Information). Similarly, when human umbilical vein endothelial cells (HUVECs) were co‐cultured with H_2_O_2_, the Cur/MOF@DS group also exhibited the most significant decline in ROS levels (Figure S8B, Supporting Information). These results illustrated the capacity of Cur/MOF@DS to specifically target SR‐A on the surface of macrophages and alleviate cellular damage resulting from oxidative stress, which reduced the formation of ox‐LDL and subsequently prevented the development of FCs, ultimately contributing to a decrease in the initiating factors associated with the onset of AS.

Macrophages can be classified into two subtypes. M1 macrophages, which dominate in progressing or vulnerable plaque, are associated with AS progression, whereas M2 macrophages, which are relatively enriched in stable plaque, are linked to the stability and regression of plaque.^[^ ^27^ ^]^ A recent research has demonstrated that impeding the release of inflammatory factors from M1 macrophages and fostering M2 macrophage polarization can effectively mitigate the inflammatory response in AS and enhance plaque stability.^[^ ^28^ ^]^ To evaluate the impact of Cur/MOF@DS on macrophage polarization, different treatments were administered following the polarization of Raw264.7 cells into M1 macrophages using LPS. Immunofluorescence staining (Figure 3E; Figure S9, Supporting Information) and flow cytometry (Figure 3F,G) exhibited a decrease in the expression of cluster of differentiation 86 (CD86, M1 macrophage marker) and an increase in the expression of mannose receptor (CD206, M2 macrophage marker) after the administration of different treatments, especially in the Cur/MOF@DS group.

In addition, AS, as a chronic inflammatory disease, exhibits inflammatory reactions throughout various stages of its development.^[^ ^29^ ^]^ Compared with stable plaques, inflammatory cell infiltration is more prominent in vulnerable plaques. Inflammatory cells, such as macrophages and T cells, aggregate in the plaque, leading to thinning of the fibrous cap, plaque calcification, and neovascularization, ultimately resulting in plaque rupture.^[^ ^30^ ^]^ Tumor necrosis factor‐alpha (TNF‐α), monocyte chemoattractant protein‐1 (MCP‐1), and interleukin‐1 beta (IL‐1β) are inflammatory cytokines closely associated with AS.^[^ ^31^ ^]^ An enzyme linked immunosorbent assay (ELISA) was employed to evaluate the levels of the three inflammatory factors in various groups following LPS stimulation of Raw264.7 cells. The results indicated that the Cur/MOF@DS group exhibited the most significant reduction in the levels of these three inflammatory factors (Figure 3H). In summary, these results indicated that Cur/MOF@DS exhibited noteworthy anti‐inflammatory and regulated macrophage polarization properties, which play a crucial role in plaque stabilization.

### 2.4 In Vitro Anti‐AS Mechanisms and Effects of Cur/MOF@DS

**Figure 4:**
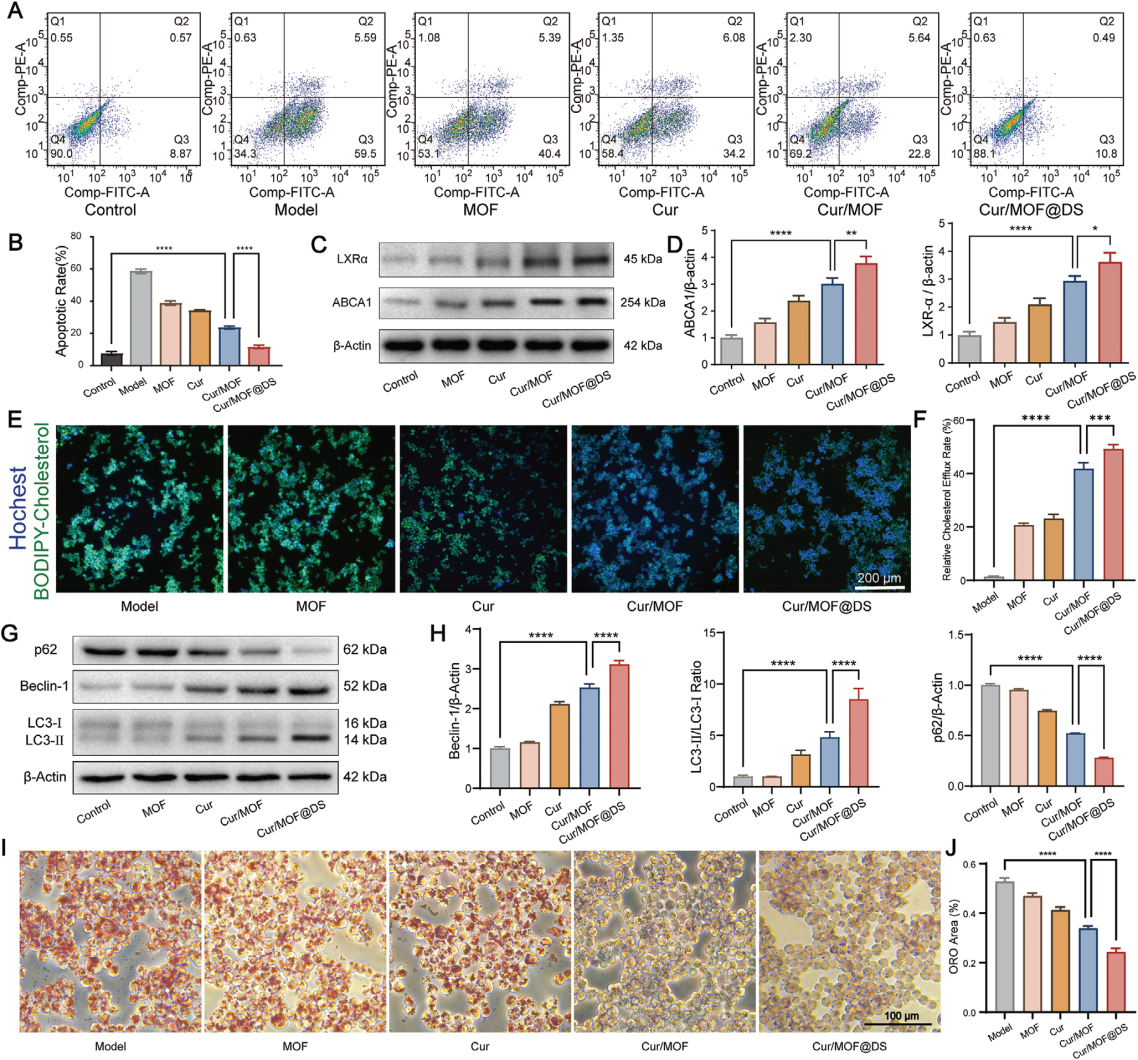
In vitro anti‐AS mechanisms and effects of Cur/MOF@DS. A) Flow cytometry quantitative analysis of Raw264.7 cells apoptosis in different treatment groups via FITC and PI staining. B) Quantitative analysis of the proportion of apoptotic cells in different treatment groups. C,D) Western blotting for cholesterol transporter proteins and quantitative analysis of macrophages subjected to different treatments. E) BODIPY‐cholesterol fluorescence intensity in cells after different treatments, following overnight co‐incubation of BODIPY‐cholesterol with Raw264.7 cells. Blue fluorescence represents Hoechst‐stained nuclei, and green fluorescence represents BODIPY‐cholesterol. F) Quantitative analysis of the relative cholesterol efflux rate in different treatment groups. G,H) Western blotting for autophagy‐related proteins and quantitative analysis of macrophages under different treatment conditions. I,J) ORO staining images and quantitative analysis of FCs co‐cultured with different treatments. Data were presented as means ± SD (*n* = 3). *P < 0.05, **P < 0.01, ***P < 0.001, ****P < 0.0001.

Under normal circumstances, ROS production and scavenging in the body maintains a dynamic balance. However, when certain pathogenic factors disrupt the balance, an increase in ROS production and/or a decrease in ROS scavenging can lead to elevation of lipid peroxidation levels in body tissues, which can cause oxidative damage to DNA and induce cells apoptosis.^[^ ^32^ ^]^ In this study, the protective role of Cur/MOF@DS against H_2_O_2_‐induced apoptosis was evaluated. Apoptosis in Raw264.7 cells was induced by H_2_O_2_ and subsequently detected by flow cytometry following Annexin V‐FITC/PI staining. The results showed that H_2_O_2_‐induced apoptosis in the Model group was significantly higher than in the Control group, whereas the number of apoptotic cells markedly decreased following the addition of Cur/MOF@DS (**Figure** **4**A,B). The above results indicated that Cur/MOF@DS can effectively attenuate the apoptotic effects induced by H_2_O_2_, thus mitigating macrophage cell death caused by oxidative stress.

Cholesterol efflux plays a crucial role in maintaining the equilibrium of cholesterol in macrophages, which is essential for preventing the accumulation of intracellular cholesterol, the formation of FCs and the development of AS.^[^ ^33^ ^]^ The LXRα/ABCA1 pathway is a crucial signaling pathway that regulates cholesterol metabolism. Within this pathway, ATP‐binding cassette transporter A1 (ABCA1) plays a key role in mediating the efflux of cholesterol. Meanwhile, Liver X receptor alpha (LXRα) can induce the expression of the ABCA1 gene to promote cholesterol efflux.^[^ ^34^ ^]^ In this study, we investigated the impact of Cur/MOF@DS on the expression of cholesterol transporter proteins in macrophages by western blotting. The results demonstrated that Cur/MOF@DS could significantly enhance the expression of LXRα and ABCA1 proteins (Figure 4C,D), suggesting that Cur/MOF@DS may increase the expression of cholesterol transporter proteins via the LXRα/ABCA1 signaling pathway, which ultimately regulates cholesterol metabolism. Furthermore, we employed the BODIPY‐cholesterol assay to directly evaluate the impact of Cur/MOF@DS on cholesterol efflux. By co‐incubating BODIPY‐cholesterol with Raw264.7 cells overnight and subsequently applying different treatments, the fluorescence intensity of BODIPY‐cholesterol within cells revealed that Cur/MOF@DS could significantly promote cholesterol efflux (Figure 4E,F; Figure S10, Supporting Information).

Additionally, macrophage autophagy also plays a significant regulatory role in AS. Studies have shown that autophagy can regulate cholesterol efflux from macrophages and prevent the formation of FCs.^[^ ^35^ ^]^ Furthermore, a recent study has demonstrated that a modest elevation in macrophage autophagy could lead to a reduction in intracellular lipid accumulation and the suppression of oxidative stress responses, which contributes to the stability of vulnerable plaques in AS.^[^ ^36^ ^]^ Beclin‐1, LC3 and p62 are important proteins that are involved in autophagy. The expression of Beclin‐1 and the activation of autophagy are positively correlated,^[^ ^37^ ^]^ and the LC3‐II/LC3‐I ratio serves as an indicator of the extent of autophagy activation.^[^ ^38^ ^]^ In contrast, p62 expression exhibited an inverse correlation with autophagy activation.^[^ ^39^ ^]^ Herein, western blotting was performed to assess the expression of these proteins. Notably, autophagy protein expression was significantly increased in the Cur/MOF@DS group (Figure 4G,H), suggesting that this nanoplatform may attenuate lipid peroxidation and inflammatory responses by promoting autophagy in macrophages. Thus, it may contribute to the stabilization of plaques and decrease the risk of acute cardiovascular events caused by plaque rupture.

FCs, the primary constituents of AS plaque, play a crucial role in disease progression.^[^ ^40^ ^]^ Inhibiting FCs formation not only impedes AS progression but also fosters plaque stability, diminishing the likelihood of acute cardiovascular events.^[^ ^41^ ^]^ The FC model was constructed by co‐culturing ox‐LDL with macrophages, and lipid droplet accumulation in FCs was detected using oil red O (ORO) staining. The results indicated that the Cur/MOF@DS group exhibited the lowest ORO staining area, which implied that Cur/MOF@DS effectively inhibited FCs formation and intracellular lipid accumulation (Figure 4I,J). In summary, the results presented above demonstrated that Cur/MOF@DS can achieve treatment effects on AS by inhibit oxidative stress‐induced macrophage apoptosis, promote macrophage autophagy, and increase the expression of cholesterol transporters.

Notably, in vitro experiments revealed markedly superior treatment efficacy in the Cur/MOF@DS group compared to the MOF@DS group. This superiority is likely attributable to the targeted delivery ability of the DS. Additionally, both Cur/MOF@DS and MOF@DS groups exhibited better treatment effects than those of the groups treated with MOF or Cur alone. This may be credited to the collaborative action of MOF and Cur within the nanoplatform, synergistically enhancing the treatment effectiveness against AS.

### 2.5 In Vivo Anti‐AS Effect and MRI of Cur/MOF@DS

**Figure 5:**
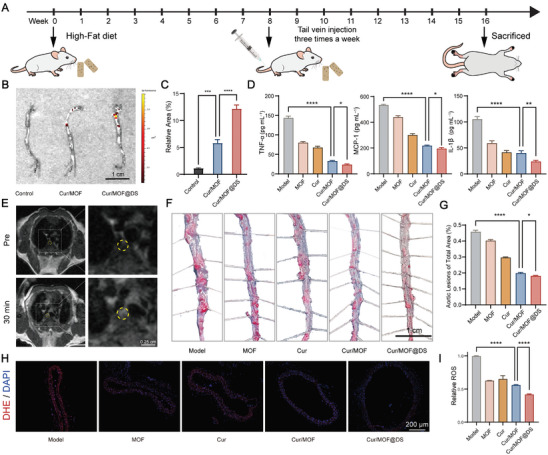
In vivo anti‐AS effects and MRI of Cur/MOF@DS. A) Schematic diagram of the treatment regimen for ApoE^−/−^ mice. B) Ex vivo fluorescence imaging of the aorta bulk after tail vein injection of FITC‐labeled nanoparticles. C) Quantitative analysis of the fluorescent area within the aorta among different treatment groups. D) Levels of serum inflammatory factors (TNF‐α, MCP‐1, IL‐1β) after different treatments. E) T1‐weighted MRI of the aorta before and after tail vein injection of Cur/MOF@DS after 30 min. The areas shown in the circle were contrast‐enhanced regions of AS plaque on MRI. F,G) ORO staining and quantitative analysis of aortic bulk after treatment. H) ROS fluorescence staining of aortic tissue frozen section in different groups. Red fluorescence represented ROS labeled with DHE, while blue fluorescence corresponded to nuclei labeled with DAPI. I) Quantitative analysis of ROS staining on frozen sections of aortic tissue. Data were presented as means ± SD (*n* = 5). *P < 0.05, ***P < 0.001, ****P < 0.0001.

Apolipoprotein E‐deficient (ApoE^−/−^) mice were used to establish AS models, and the treatment effects of Cur/MOF@DS on AS were investigated. 8‐week‐old male ApoE^−/−^ mice were divided into five groups (**Figure** **5**A). The animals were fed with a high‐fat diet for 8 weeks to establish an AS animal model, and then treated with Cur/MOF@DS for 8 weeks to evaluate its treatment effect. To confirm in vivo plaque targeting, FITC‐labeled Cur/MOF@DS was injected intravenously via the tail vein. Ex vivo fluorescence imaging of the aorta revealed notable accumulation of the nanoplatform in the plaque region with DS modification (Figure 5B,C). Subsequently, the levels of inflammatory factors in the serum of the mice in each group were quantified. The results showed a reduction in the levels of the three inflammatory factors in all groups compared to the Model group (Figure 5D), with the Cur/MOF@DS group exhibiting the most notable reduction. Moreover, the in vivo MRI performance of the nanoplatform was assessed. Following the administration of the nanoplatform via the tail vein, MRI revealed a significantly high signal in the aortic region for both Cur/MOF@DS and Cur/MOF groups (Figure 5E; Figure S11A,B, Supporting Information). However, the Cur/MOF@DS group exhibited superior imaging quality compared to the Cur/MOF group. Quantitative analysis of the gray values in the contrast‐enhanced regions further confirmed its suitability for vascular MRI (Figure S11C, Supporting Information). This finding may provide a novel approach for plaque MRI and composition analyses. In addition, the plaque areas of the aortic bulk (Figure 5F,G) and the lipid content of the aortic arch section (Figure S12, Supporting Information) were evaluated using ORO staining. These results indicated that Cur/MOF@DS substantially minimized the plaque area and exhibited the most effective anti‐AS effect. Finally, a fluorescent probe, dihydroethidium (DHE), was used to detect ROS in aortic frozen sections. The results indicated that the aortic tissue in the Cur/MOF@DS group exhibited the lowest average fluorescence intensity in red (Figure 5H,I; Figure S13, Supporting Information), implying that Cur/MOF@DS could effectively eliminates ROS within the local plaque microenvironment.

### 2.6 In Vivo Evaluation of AS Plaque Stability after Cur/MOF@DS Treatment

**Figure 6:**
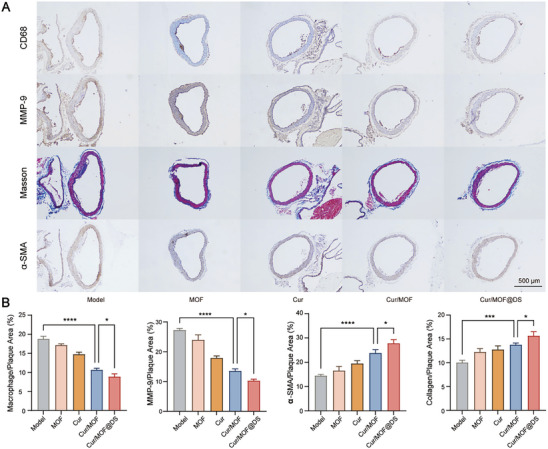
In vivo evaluation of AS plaque stability after Cur/MOF@DS treatment. A,B) Masson staining, CD68 antibody staining, MMP‐9 antibody staining and α‐SMA antibody staining of aortic arch sections with quantitative analysis. Data were presented as means ± SD (*n* = 5). *P < 0.05; **P < 0.01; ****P < 0.0001.

The composition of AS plaque profoundly impacts plaque stability, which is strongly correlated with the occurrence of acute cardiovascular events.^[^ ^42^ ^]^ Cluster of differentiation 68 (CD68), which is widely expressed on macrophages, serves as a significant biomarker for macrophage identification within plaques region.^[^ ^43^ ^]^ Likewise, Matrix metalloproteinase 9 (MMP‐9) can degrade collagen, elastin and other extracellular matrix components within the plaque, which can ultimately lead to plaque disruption and hemorrhage.^[^ ^44^ ^]^ The immunohistochemical staining results of CD68 and MMP‐9 showed a significant reduction in macrophage infiltration and MMP‐9 expression within the plaque region, which is favorable for plaque stability (**Figure** **6**A,B). In contrast, Masson staining highlights the intricate distribution of collagen fibers and serves as a valuable tool for assessing collagen content within AS plaques.^[^ ^45^ ^]^ Similarly, alpha‐smooth muscle actin (α‐SMA), a marker of smooth muscle cells differentiation, could provide insights into the thickness of the fibrous cap within the plaque, which is associated with plaque stability as well.^[^ ^46^ ^]^ The results of Masson staining and α‐SMA immunohistochemistry indicated that after treatment with Cur/MOF@DS, there was a notable increase in the amount of collagen fibers and vascular smooth muscle within the plaque region. This development was advantageous for enhancing plaque stability and reducing the incidence of ASCVD resulting from plaque rupture.

Notably, in the in vivo experiments, Cur/MOF@DS exhibited a significant treatment effect in treating AS, surpassing the effects observed in the MOF@DS, MOF or Cur groups. This outcome aligns with the in vitro experimental results, which further support the exceptional targeting capabilities of DS, as well as the synergistic action of MOF and Cur in enhancing AS treatment.

### 2.7 Transcriptomic Study in ApoE^−/−^ after Cur/MOF@DS Treatment

**Figure 7:**
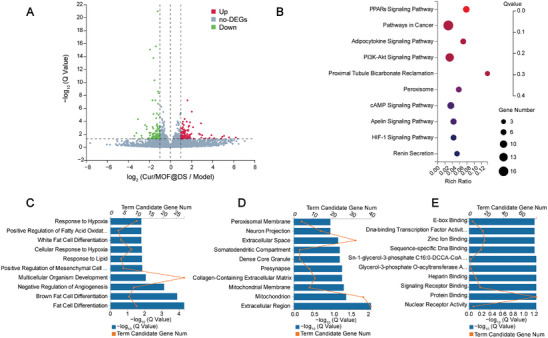
Transcriptomic study of ApoE^−/−^ mice after Cur/MOF@DS treatment. A) Volcano plot showing the number of up‐ and down‐regulated genes in the Cur/MOF@DS group compared with the Model group. The X‐axis represented the fold change of the difference after conversion to log2, and the Y‐axis represented the significance value after conversion to ‐log10. Red represented upregulated DEGs, blue represented downregulated DEGs, and gray represented non‐DEGs. B) KEGG pathway enrichment analysis of DEGs between the two groups. C−E) GO enrichment analyses of DEGs in two groups: BP, CC, and MF.

To further elucidate the mechanism of Cur/MOF@DS in treating AS, a transcriptomic analysis was conducted to evaluate the differences in gene expression between the Cur/MOF@DS and Model groups. As shown in the volcano plot (**Figure** **7**A), 222 significantly differentially expressed genes (DEGs) were identified, including 107 upregulated and 115 downregulated genes. The Kyoto Encyclopedia of Genes and Genomes (KEGG) results (Figure 7B) highlighted DEGs mainly enriched in pathways related to peroxisome proliferator‐activated receptors (PPARs), adipocytokine, and phosphoinositide 3‐kinase (PI3K)/Akt signaling pathways. PPARs, nuclear hormone receptors activated by fatty acids and their derivatives, play a pivotal role in regulating plasma cholesterol and triglyceride levels, thereby influencing cholesterol homeostasis within macrophages and the progression of AS.^[^ ^47^ ^]^ The adipocytokine signaling pathway encompasses various hormones and cytokines synthesized and secreted by adipocytes, including adiponectin, adipocyte complement‐related proteins, leptin resistance hormone and apoptosis induction factor. These hormones and cytokines regulate the activity of multiple downstream signaling pathways by binding to their receptors, thereby affecting diverse physiological processes, such as energy metabolism, glucose metabolism and lipid metabolism.^[^ ^48^ ^]^ PI3K/Akt is a member of the lipid kinase family that regulates AS by participating in processes such as apoptosis, macrophage phenotype transformation, antiplatelet aggregation, anti‐inflammation, oxidative stress regulation, and autophagy.^[^ ^49^ ^]^ Gene Ontology (GO) categorizes gene functions into three main categories: biological process (BP), cellular component (CC) and molecular function (MF).^[^ ^50^ ^]^ The GO enrichment analysis unveiled involvement of various BP, including fat cell differentiation, brown fat cell differentiation, and response to lipids (Figure 7C). Additionally, in the context of CC, the results highlighted the importance of the mitochondrion and the collagen‐containing extracellular matrix (Figure 7D). Furthermore, MF, such as nuclear receptor activity, protein binding, and glycerol‐3‐phosphate O‐acyltransferase activity (Figure 7E), were found to be associated with the treatment process. In conclusion, all these processes and functions were crucial in the context of AS treatment by this nanoplatform.

### 2.8 Biosafety Assessment

The biosafety of the nanoplatforms was evaluated. In the hemolysis test (Figure S14, Supporting Information), no significant erythrocyte lysis was observed even at 200 µg mL^−1^ of MOF and Cur/MOF@DS, indicating excellent hemocompatibility. Subsequently, cell counting kit‐8 (CCK‐8) assays (Figure S15, Supporting Information) and live/dead cell viability assays (Figure S16, Supporting Information) were conducted following co‐cultivation of MOF and Cur/MOF@DS with Raw264.7 cells. These results provided additional evidence affirming the safety of the material. Finally, the in vivo safety of Cur/MOF@DS was preliminarily evaluated. During the 8 weeks treatment period, no significant changes in body weight were observed (Figure S17, Supporting Information). Similarly, no notable abnormalities were detected in the hematoxylin and eosin (H&E) sections (Figure S18, Supporting Information), blood counts (Figure S19, Supporting Information), liver and kidney function (Figure S20 Supporting Information), and lipid levels (Figure S21, Supporting Information) across major organs after treatment. In conclusion, these findings indicated that Cur/MOF@DS exhibited a favorable safety profile for intravenous administration.

## 3 Conclusion

In summary, we developed a drug delivery platform based on MOF with both nanozyme activities and MRI capabilities for the diagnosis and treatment of ASCVD. By surface modification with DS on the nanoplatform, Cur can be transported in a targeted manner to the plaque site. Subsequently, MOF and Cur worked collaboratively to scavenge oxygen free radicals and mitigate cellular damage induced by oxidative stress, leading to a reduction in ox‐LDL generation. Cell experiments demonstrated that Cur/MOF@DS facilitated cholesterol efflux through inducing macrophage autophagy, and decreased FCs formation by promoting M2 macrophage polarization. The results of animal experiments revealed that Cur/MOF@DS exhibited robust efficacy against AS and held promise for MRI applications to achieve early diagnosis. However, this nanoplatform suffered from the shortcomings of single targeting, multiple drug injections, and potential toxicity induced by metal ion accumulation during long‐term use. These issues necessitate further refinement and optimization in future studies. Prospectively, MOF, which acts as a sonosensitizer, is promising for the prevention and treatment of in‐stent restenosis in coronary arteries after stent implantation through sonodynamic therapy.

## Conflict of Interest

The authors declare no conflict of interest.

## Supporting information

Supporting Information

## Data Availability

The data that support the findings of this study are available from the corresponding author upon reasonable request.
