# Supplementary material for: Curcumin Equipped Nanozyme‐Like Metal−Organic Framework Platform for the Targeted Atherosclerosis Treatment with Lipid Regulation and Enhanced Magnetic Resonance Imaging Capability
Source: Adv Sci (Weinh). 2024 May 2;11(26):2309062. doi: 10.1002/advs.202309062 (PMC11234396; doi:10.1002/advs.202309062)
Supplement: Supplementary file 1 — Supporting Information [file ADVS-11-2309062-s001.pdf]

ADVANCED SCIENCE
Open Access

Supporting Information

for *Adv. Sci.*, DOI 10.1002/advs.202309062

Curcumin Equipped Nanozyme-Like Metal–Organic Framework Platform for the Targeted Atherosclerosis Treatment with Lipid Regulation and Enhanced Magnetic Resonance Imaging Capability

Fanzhen Lv, Huaqiang Fang, Li Huang, Qingqing Wang, Shuangyuan Cao, Wenpeng Zhao, Zhibin Zhou, Weimin Zhou and Xiaolei Wang**

Supporting Information

Curcumin Equipped Nanozyme-like Metal−Organic Framework Platform for the Targeted Atherosclerosis Treatment with Lipid Regulation and Enhanced Magnetic Resonance Imaging Capability

Fanzhen Lv, Huaqiang Fang, Li Huang, Qingqing Wang, Shuangyuan Cao, Wenpeng Zhao, Zhibin Zhou, Weimin Zhou, Xiaolei Wang**

F. Lv, H. Fang, L. Huang, W. Zhao, Z. Zhou, Prof. W. Zhou
Department of Vascular Surgery, the Second Affiliated Hospital, Jiangxi Medical College, Nanchang University, Nanchang, Jiangxi 330006, P.R. China
E-mail: drzwm@sina.com

Q. Wang
School of Pharmacy, Nanchang University, Nanchang, Jiangxi 330006, P.R. China

S. Cao
The National Engineering Research Center for Bioengineering Drugs and the Technologies, Institute of Translational Medicine, Nanchang University, Nanchang, Jiangxi 330006, P.R. China

Prof. X. Wang
School of Pharmacy, Nanchang University, Nanchang, Jiangxi 330006, P.R. China;
The National Engineering Research Center for Bioengineering Drugs and the Technologies, Institute of Translational Medicine, Nanchang University, Nanchang, Jiangxi 330006, P.R. China
E-mail: wangxiaolei@ncu.edu.cn

Keywords: atherosclerosis, targeted drug delivery, metal−organic frameworks, reactive oxygen species scavenging, magnetic resonance imaging

Experimental Section

Materials: 4-Formylbenzoic acid (98.0%), propionic acid (≥ 99.5%), pyrrole (> 99.5%), N,N-dimethylformamide (DMF, > 99.9%), ethanol (> 99.5%), manganese chloride tetrahydrate ($MnCl_2·4H_2O$, 99.9%), zirconyl chloride octahydrate ($ZrOCl_2·8H_2O$), curcumin (Cur) and dextran sulfates (DS) sodium salt (MW = 5000 Da) were purchased from Shanghai Macklin Biochemical Technology Co., Ltd. Annexin V-FITC/PI apoptosis detection kit and CCK-8 were purchased from Beyotime Biotechnology. The ROS assay kit, DAPI solution, TNF-α ELISA kit, MCP-1 ELISA kit and IL-1β ELISA kit were procured from Solarbio Co., Ltd. The BODIPY-cholesterol was procured from MedChemExpress. All the chemicals were used without further purification.

Synthesis of MOF: 2.22 g 4-Formylbenzoic acid was dissolved in 70 mL propionic acid. Subsequently, 1.02 mL of pyrrole was added dropwise to the solution, and the mixture was heated and refluxed for 2 h. The reaction mixture was then cooled to room temperature and filtered. The filter cake was washed with deionized water and dried under vacuum to obtain 5,10,15,20-tetrakis (4-carboxyphenyl) porphyrin (TCPP).

0.5 g of TCPP was mixed with 80 mL of DMF, and 2.5 g of $MnCl_2·4H_2O$ was added before heating and refluxing the mixture for 1 h. After the reaction was complete, 6.0 mol L^{-1} hydrochloric acid was slowly added dropwise until the solid was completely dissolved. Mn-TCPP was obtained via filtration, washing and drying.[1]

Finally, 30 mg $ZrOCl_2·8H_2O$ and 280 mg benzoic acid were sonicated to dissolve, then the mixture was incubated in an oven at 80°C for 1 h. After cooling to room temperature,10.98 mg Mn-TCPP was introduced and subjected to sonication for 5 min until fully dissolved. The reaction was performed in an oil bath at 120°C for 1 h, followed by cooling, centrifugation, washing, and drying to obtain MOF.[2]

Synthesis of Cur/MOF@DS: 5 mg MOF was ultrasonically dissolved in 5 mL DMF. Subsequently, 1.5 mg Cur was added to the solution at room temperature and stirred overnight in darkness. Then, the mixture was centrifuged at 8000 rpm for 10 min. After washing and drying, Cur/MOF was obtained.

The Cur/MOF was dispersed to form a 1 mg mL^{-1} Cur/MOF solution, the dissolved DS was added to the Cur/MOF solution. The mixture was stirred overnight at room temperature to obtain Cur/MOF@DS via electrostatic adsorption.

Characterization of MOF: The morphology of MOF and Cur/MOF@DS were analyzed by SEM (ZEISS/Sigma300, Germany) and TEM (Inspect F50, US). In addition, the EDS spectrum of MOF was also obtained. XPS measurements were performed using an ESCALAB 220iXL

spectrometer. The absorption spectra of the materials were measured using a UV−vis spectrophotometer (UV-2600, Japan). The pore sizes of MOF were measured using a thermogravimetric analyzer (Discovery TGA 55612-1, USA). The particle size and Zeta potentials of the material were determined using a Particle Size and Zeta Potential Analyzer (Nano ZS 90020502, UK). The FTIR spectroscopy was performed on a Nicolet iS5 FTIR Spectrometer.

In Vitro Drug Loading: 5 mg of MOF was dispersed in 5 mL DMF, and different masses of Cur were added to achieve the mass ratios of Cur to MOF at 0.1:1.0, 0.2:1.0, 0.4:1.0, 0.6:1.0 and 0.8:1.0, respectively. The mixture was stirred overnight in darkness at room temperature. Subsequently, each mixed solution was collected and centrifuged at 8000 rpm for 10 min. The Cur content in the supernatant was determined by UV−vis at the wavelength of 426 nm to calculate the drug loading capacity of MOF for each concentration ratio.

$$\text{Drug loading content} = \frac{W_0 - W_1}{W_2} \times 100\% \qquad \text{Equation (1)}$$

W_0 is the total mass of added Cur, W_1 is the mass of Cur in the supernatant, and W_2 is the total mass of the nanoplatform.

In Vitro Drug release efficiency: 5 mg of Cur/MOF@DS powder was dissolved in 5 mL phosphate buffered saline (PBS). Then, centrifuge tubes containing the solution were placed in a water bath at 37°C in darkness. At intervals of 6, 12, 24, 36 and 48 h, one set of centrifuge tubes was removed, and the Cur content in the supernatant was measured.

$$\text{Drug release rate (\%)} = \frac{W_1}{W_0} \times 100\% \qquad \text{Equation (2)}$$

W_1 is the Cur content in the supernatant, and W_0 is the drug loading of the nanoplatform.

ROS Scavenging Test: The catalase-like activity was evaluated using UV−vis spectroscopy to assess the ability of different concentrations of MOF to remove H_2O_2. Different concentrations (0, 30, 60, 125, 250, and 500 μg mL^{-1}) of aqueous MOF and Cur/MOF@DS solutions were rapidly mixed with 150 μL of 1 mol L^{-1} H_2O_2 solution. After a 30 min reaction, 1 mol L^{-1} potassium iodide (KI) solution was added, and the absorbance of the mixture was measured at 350 nm to calculate the H_2O_2 clearance efficiency. Next, as hydrogen peroxide can oxidize TMB to produce a color reaction, the peroxidase-like activity of MOF and Cur/MOF@DS was evaluated by measuring the absorbance change of the solution at 650 nm wavelength after different reaction times using 0.1 mM TMB. Furthermore, different concentrations of MOF and Cur/MOF@DS solution were mixed with 1 mM DPPH free radical scavengers, and the absorbance of the mixture at 517 nm was measured to assess the free radical scavenging ability of MOF. Finally, TiO_2 was employed to simulate the generation of hydroxyl and superoxide

anions, and DMPO was chosen as the capturing agent. The scavenging capability of PCN-222 on hydroxyl and superoxide anions was assessed utilizing ESR.

Magnetic Resonance Imaging Test of MOF: The manganese (Mn) ion content in MOF and Cur/MOF@DS were quantified using ICP-OES. Subsequently, MOF and Cur/MOF@DS were prepared in solutions containing different concentrations of Mn ion (0.00, 0.03, 0.06, 0.13, 0.25, and 0.50 mmol L^{-1}).

T1 relaxation time, T1-weighted images of MOF were acquired on a 3.0 T MRI scanner (Siemens Prisma 3.0 T MR scanner, Siemens Healthineers, Erlangen, Germany) with the following parameter: T1, inversion recovery sequence, repetition time = 12000 ms, inversion time = 20, 40, 80, 160, 320, 640, 1280 and 2560 ms.

T1 relaxation time, T1-weighted images of Cur/MOF@DS were acquired on a 1 T MRI scanner (NM42-040H-I Small Animal MRI System, Suzhou Niumag Analytical Instrument Corporation, Suzhou, China) with the following parameters: T1, spin echo sequence, repetition time = 495 ms, echo time = 20 ms, matrix = 256 × 256, number of acquisition = 6.

Hemolysis Assay: The obtained blood was centrifuged three times with physiological saline (1500 rpm, 15 min) to obtain pure red blood cells (RBC). Subsequently, 0.1 mL of RBC was added to 0.9 mL of ultrapure water, physiological saline, and physiological saline containing different concentrations of MOF (12, 25, 50, 100 and 200 μg mL^{-1}), respectively. After incubation at 37°C for 1 h, low-speed centrifugation was performed, and the absorbance of the supernatant in each group was measured at a wavelength of 540 nm.

$$\text{Hemolysis rate (\%)} = \frac{I - I_{NS}}{I_0 - I_{NS}} \times 100\% \qquad \text{Equation (3)}$$

I represent the absorbance of the supernatant from MOF at different concentrations, I_{NS} represents the absorbance of the supernatant from the physiological saline group, and I_0 represents the absorbance of the supernatant from the ultrapure water group.

Live/Dead Cell Staining Assay: HUVECs and Raw264.7 cells were evenly seeded in a 24-well plate at a density of 5 × 10^4 cells/well and cultured for 12 h. The culture medium was then replaced with Dulbecco's Modified Eagle Medium (DMEM) containing varying concentrations of MOF (0, 12.5, 25, 50, 100 and 200 μg mL^{-1}), and the cells were further cultured for 24 h. After the cells were washed with PBS, Calcein-AM/PI was added to the cell samples and incubated in darkness for 15 min.

Cell Compatibility: CCK-8 assay was used to assess the cytotoxicity of different concentrations of MOF and Cur/MOF@DS. Raw264.7 cells were seeded in a 96-well microplate (1 × 10^4 cells/well) and cultured for 12 h. Subsequently, the cells were incubated with serum-free culture medium containing different concentrations of MOF and Cur/MOF@DS (0, 12.5, 25, 50, 100

and 200 μg mL^{-1}) for 24 and 48 h. Then, 10 μL of the CCK-8 solution was added to each well and incubated for 2 h. The absorbance of each sample was measured using a microplate reader at the wavelength of 450 nm. Cells cultured without CCK-8 and MOF were designated as the blank group, and their cell survival rate was set at 100%.

$$\text{Cell viability (\%)} = \frac{OD_1 - OD_0}{OD_2 - OD_0} \times 100\% \qquad \text{Equation (4)}$$

OD_1 is the optical density (OD) of the experimental group, OD_2 is the OD of the Control group, and OD_0 is the OD of the blank group.

In Vitro Targeting Capability: FITC-labeled Cur/MOF and Cur/MOF@DS were synthesized according to the method previously described, which were then co-cultured with Raw264.7 cells pre-treated with 1 μg mL^{-1} LPS for 3 h. In addition, the Raw264.7 cells membranes were labeled with DID fluorescent dye. Subsequently, colocalization of FITC and DID fluorescence was observed under an inverted fluorescence microscope.

In Vitro ROS Scavenging: The Raw264.7 cells were divided into five groups: Control, Model, MOF, Cur, Cur/MOF and Cur/MOF@DS. Cells were seeded into 24-well plates at a density of 5×10^5 cells per well and incubated for 12 h. Subsequently, the medium in each group was replaced with serum-free medium containing 100 ng mL^{-1} of different materials and incubated for 2 h. Except for the Control group, each group was further incubated with serum-free medium containing 1 μg mL^{-1} LPS for 4 h. Finally, the cells were cultured in serum-free medium supplemented with DCFH-DA (1:1000) and Hoechst (500 ng mL^{-1}) for 20 min in darkness, and observed using an inverted fluorescence microscope. HUVECs were induced with 1 μmol L^{-1} H_2O_2, and the remaining procedures were similar to those described above.

Immunofluorescence and Flow Cytometry of Macrophage Polarization: The Raw264.7 cells were divided into Control, Model, MOF, Cur, Cur/MOF and Cur/MOF@DS groups, and corresponding pretreatments were performed. Subsequently, each group was stimulated with 100 ng mL^{-1} LPS for 24 h to induce inflammation for subsequent analysis. A portion of the cells was used to prepare cell slides for immunofluorescence, which were incubated with primary and secondary antibodies (Anti-CD86 antibody, Anti-CD206 antibody) and DAPI staining solution. Another portion of the cells was collected and incubated with primary and secondary antibodies (Anti-CD86 antibody, Anti-CD206 antibody) for flow cytometry analysis.

ELISA Analysis: Raw264.7 cells were cultured in a 24-well plate and preincubated with serum-free DMEM containing different materials for 6 h. Except for the Control group, which received treatment with serum-free DMEM alone, the cells in the other groups were incubated with serum-free DMEM containing ox-LDL (50 μg mL^{-1}) for 24 h. After treatment, the cell culture supernatants were collected and ELISA was performed according to the instructions. The OD

values of each group were measured at 450 nm, and the expression levels of inflammatory factors (TNF-α, MCP-1 and IL-1β) in the supernatant were calculated based on the OD values.

Antioxidation Activities Explorations: Raw264.7 cells were seeded in 12-well plates at a density of 3 × 10^5 cells per well and incubated for 12 h. Following this, the culture medium for all groups, except for the Control group, was replaced with medium containing different materials at a concentration of 100 μg mL^{-1} and incubated for an additional 2 h. Subsequently, the medium of the Control group was replaced with medium containing 100 μmol L^{-1} H_2O_2 and incubated for another 12 h to induce apoptosis. The apoptosis inhibition results were obtained using a flow cytometer according to the instructions.

Expression of cholesterol transport-related proteins and macrophage autophagy-related proteins: Raw264.7 cells were seeded at a density of 1 × 10^5 cells per mL in a 24-well cell culture plate and incubated overnight. The culture medium was then replaced with fresh medium containing different materials and incubated for 24 h. Total protein was extracted from the cells, and the protein concentration was determined. 20 μg of protein from each group was separated using sodium dodecyl sulfate-polyacrylamide gel electrophoresis (SDS-PAGE) and transferred to polyvinylidene fluoride (PVDF, IPVH00010, Millipore) membranes. Subsequently, the membranes were blocked with 5% bovine serum albumin (BSA) at room temperature for 1 h, followed by incubation overnight at 4℃ with anti-LXRα, anti-ABCA1, anti-Beclin-1, anti-LC3-I, anti-LC3-II, anti-p62 and anti-β-actin antibodies Subsequently, the membranes were incubated with the secondary antibodies at 37℃ for 60 min. Protein bands were visualized using a ChemiDoc XRS+ integrated chemiluminescence imaging system (Bio-Rad) and quantified using the ImageJ software.

Cholesterol Efflux Assay: Raw264.7 cells were cultured at a density of 1×10^5 cells/well in 24-well plates overnight and were then replaced with serum-free medium containing 10 μM BODIPY-cholesterol to continue incubation for 12 h. Following this, the cells were washed twice with serum-free DMEM and then co-incubated with serum-free medium alone or containing different drugs (100 ng mL^{-1}) for 12 h. After staining with DAPI staining solution, the cells were observed under an inverted fluorescence microscope and the quantitative analysis of fluorescence intensity (FI) was carried out using ImageJ software. Cells without drugs treatment was designated as the Model group.

$$\text{Relative Cholesterol Efflux Rate (\%)} = \frac{FI_0 - FI_1}{} \times 100\% \qquad \text{Equation (5)}$$

FCs ORO Staining: The cells were incubated in serum-free DMEM with different samples for 6 h, followed by incubation with serum-free DMEM containing ox-LDL at a concentration of 50 μg mL^{-1} for 24 h to induce foam cell formation. The cells were fixed with 4% paraformaldehyde for 30 min, and then stained with ORO according to the instructions.

AS Animal Model: All animal experiments were conducted in compliance with the principles of the care and use of laboratory animals. All procedures were approved by the Institutional Animal Care and Use Committee of Nanchang University (Approval No. NCULAE-20221228017). Eight-week-old male apolipoprotein E-deficient ($ApoE^{-/-}$) mice purchased from Hangzhou Ziyuan Experimental Animal Technology were selected and fed a high-fat diet to establish the AS model.

In Vivo Plaque Targeting and MRI: After 8 weeks of high-fat diet feeding, FITC-labeled Cur/MOF@DS was intravenously injected into $ApoE^{-/-}$ mice via the tail vein (100 μg kg^{-1}). After 8 h, the mice were anesthetized for heart perfusion, and the aorta was isolated for *ex vivo* fluorescence imaging.

The *in vivo* MRI of the Cur/MOF and Cur/MOF@DS were determined using an $ApoE^{-/-}$ mouse model on a 3.0 T MR scanner (MRS-3000, MR Solution, Guildford, UK). MR scans were conducted before tail vein injection and 1 h after injection (100 μL). The following parameters were used to monitor MRI under the T_1 model at different time points: slice thickness of 3 mm, field of view of 60 mm, repetition time of 340 ms, and echo time of 16 ms.

In Vivo Anti-AS Effect: Eight-week-old $ApoE^{-/-}$ mice were randomly divided into five groups (**Figure 5**A) using the random number table method, and then fed with a high-cholesterol diet for 8 weeks to induce AS models. Thereafter, the mice received intravenous injections of various materials thrice weekly over a period of 8 weeks (10 mg kg^{-1}), and their body weight was monitored throughout the treatment regimen. At the end of the treatment, the mice were anesthetized, and blood plasma was collected for biochemical analysis. Subsequently, the mice were euthanized using cervical dislocation. Aortic tissues were isolated for ROS staining of frozen section experiment, hematoxylin and eosin (H&E) staining, immunohistochemical analysis, and ORO staining of the aortic arch. Additionally, major organs (heart, liver, spleen, lung and kidney) were subjected to H&E staining to evaluate the safety of the nanoplatform.

Transcriptome Analysis: Following the conclusion of the treatment, aortas bulk from both the Model and Cur/MOF@DS groups were collected and promptly frozen in liquid nitrogen. High-

throughput sequencing was conducted by BGI Genomics, and the data were analyzed using the Dr.Tom multi-omics data mining system online.

Statistical Analysis: All experiments were repeated a minimum of three times, and data were presented as mean ± SD. Statistical analysis was performed using GraphPad Prism 9.4.1 software (GraphPad Software, USA), utilizing two-tailed *t*-tests and one-way analyses of variance (ANOVA). Statistical significance was denoted as * for $P < 0.05$, ** for $P < 0.01$, *** for $P < 0.001$, and **** for $P < 0.0001$.

References

[1] Y. Jiang, T. Su, Z. Qin, G. Huang, *RSC Adv.* **2015**, *5*, 24788.
[2] Q. Xu, G. Zhan, Z. Zhang, T. Yong, X. Yang, L. Gan, *Theranostics* **2021**, *11*, 1937.

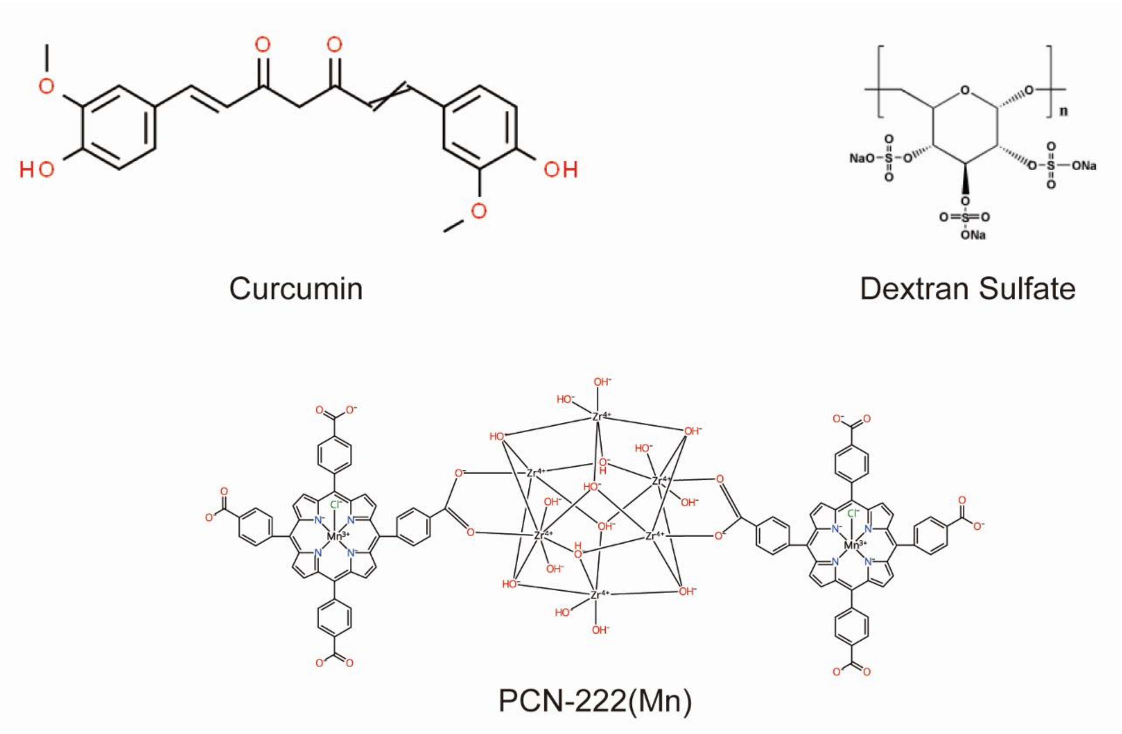

Figure S1. The chemical structures of Cur, DS and MOF.

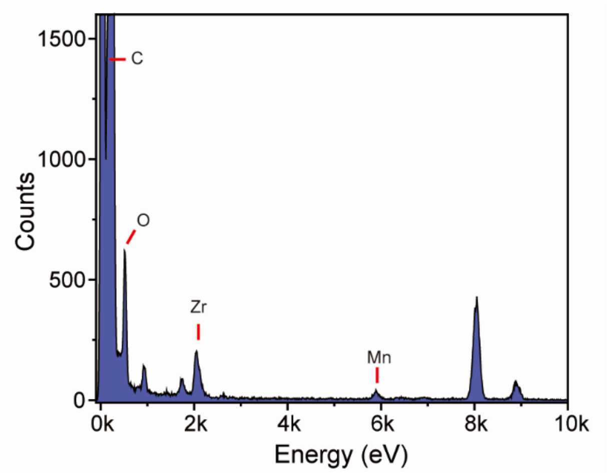

Figure S2. EDS spectrum of MOF.

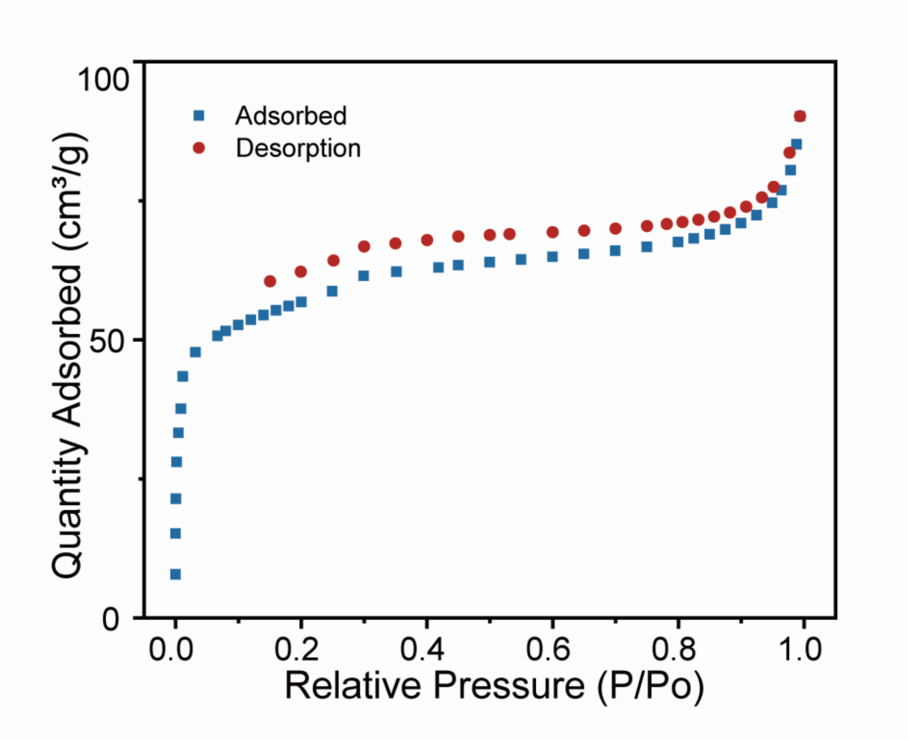

Figure S3. Nitrogen adsorption and desorption isotherms at 77 K of Cur/MOF@DS.

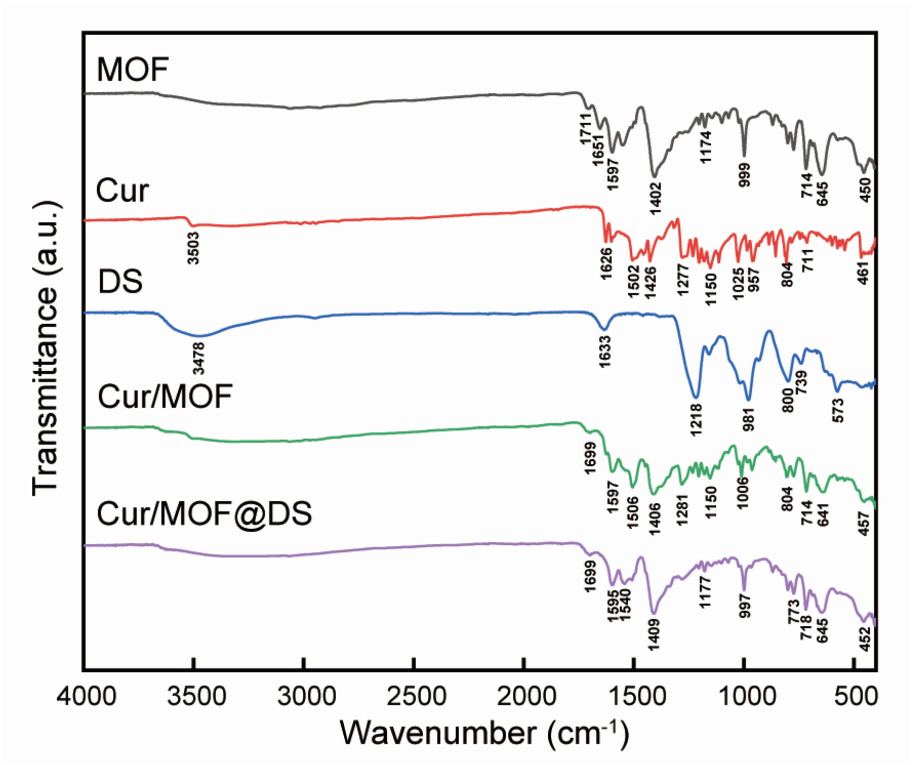

Figure S4. FTIR spectra of MOF, Cur, DS, Cur/MOF and Cur/MOF@DS.

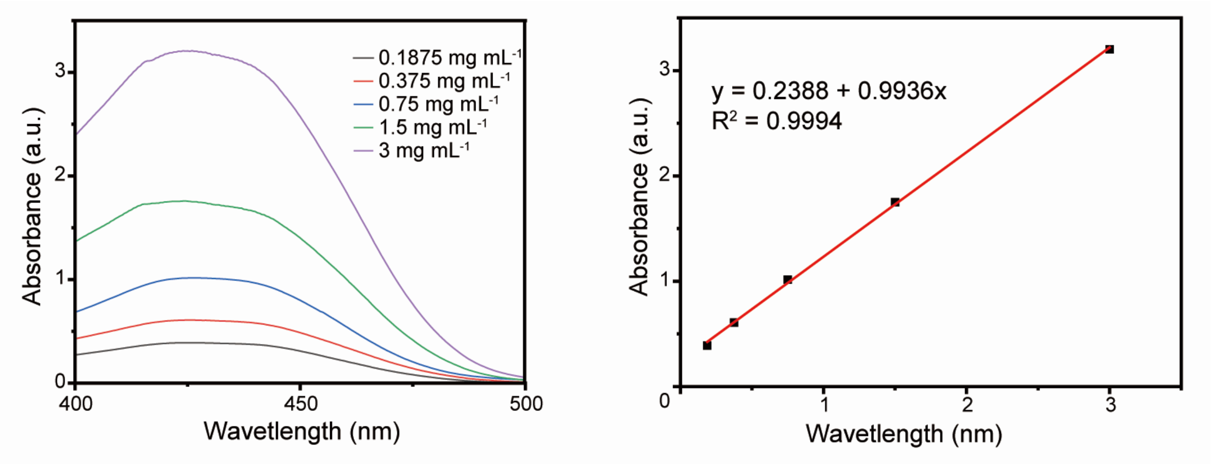

Figure S5. A) UV–vis Absorption Spectra of Cur/DMF Solutions with Different Concentrations. B) Standard Curve of Cur/DMF Solutions. Data are presented as means ± SD.

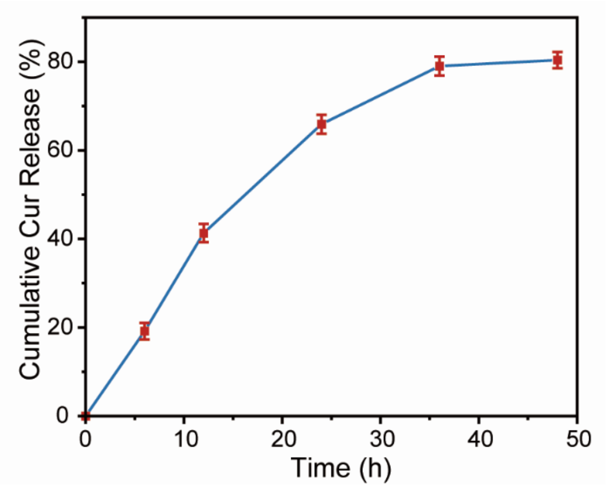

Figure S6. Cur release curve of Cur/MOF@DS *in vitro*. Cur concentrations in the supernatant detected by UV−vis at 6, 12, 24, 36 and 48 h. Data are presented as means ± SD (n = 3).

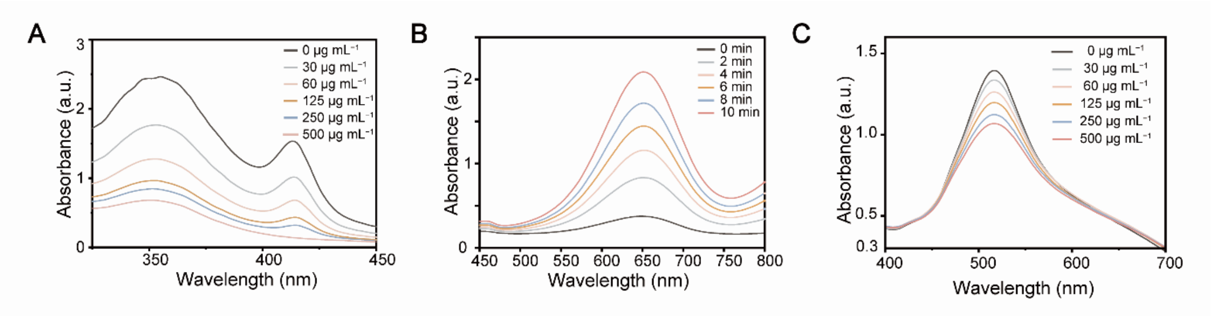

Figure S7. A) Evaluation of hydrogen peroxide scavenging ability at varying MOF concentrations in 1 mM H_2O_2 and 1 M potassium iodide (KI) solution. B) Evaluation of peroxide scavenging ability after different reaction time by mixed MOF and 0.1 mM TMB with 1 mM H_2O_2 solution. C) Evaluation of DPPH radical scavenging ability at different MOF concentrations in 1 mM DDPH free radical scavenger solution.

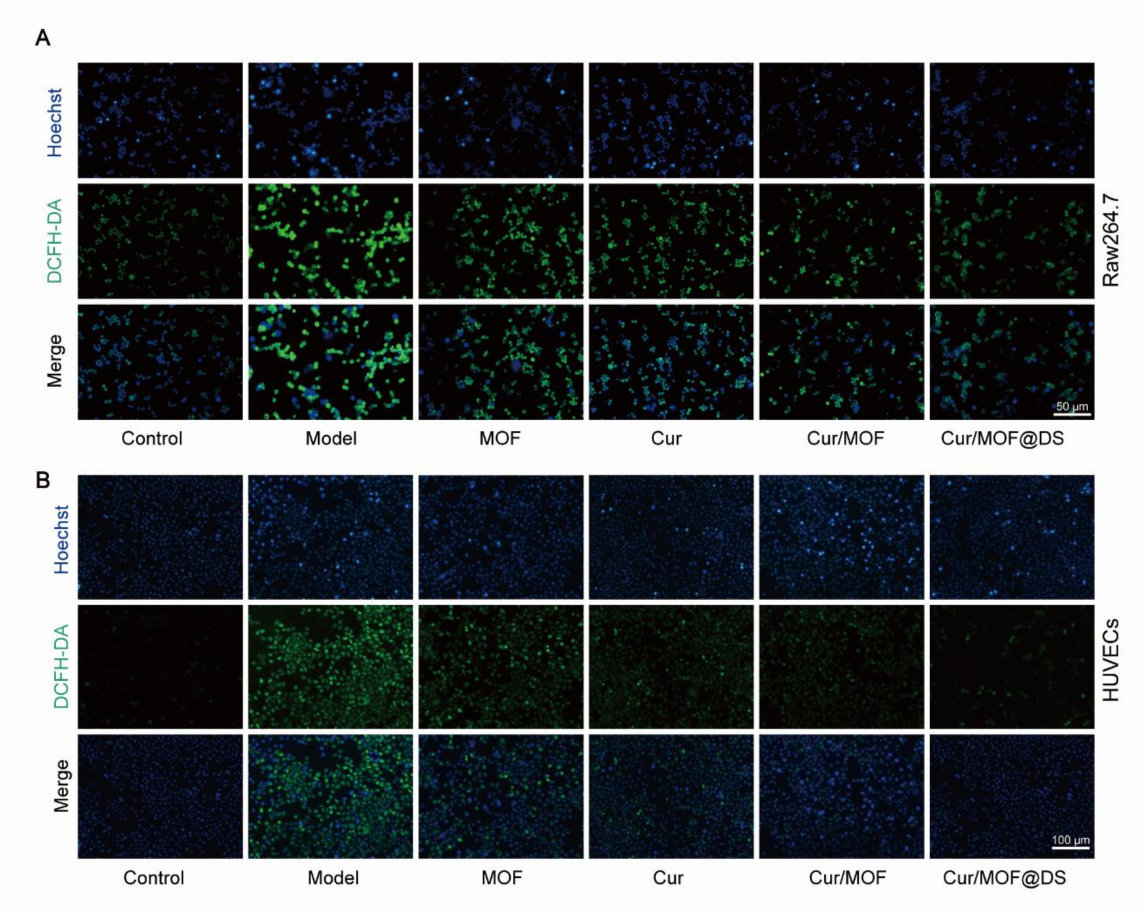

Figure S8. ROS fluorescence of Raw264.7 cells (A) and HUVECs (B) after treatment with different materials. Blue indicated Hoechst-stained nuclei, and green indicated ROS stained by DCFH-DA.

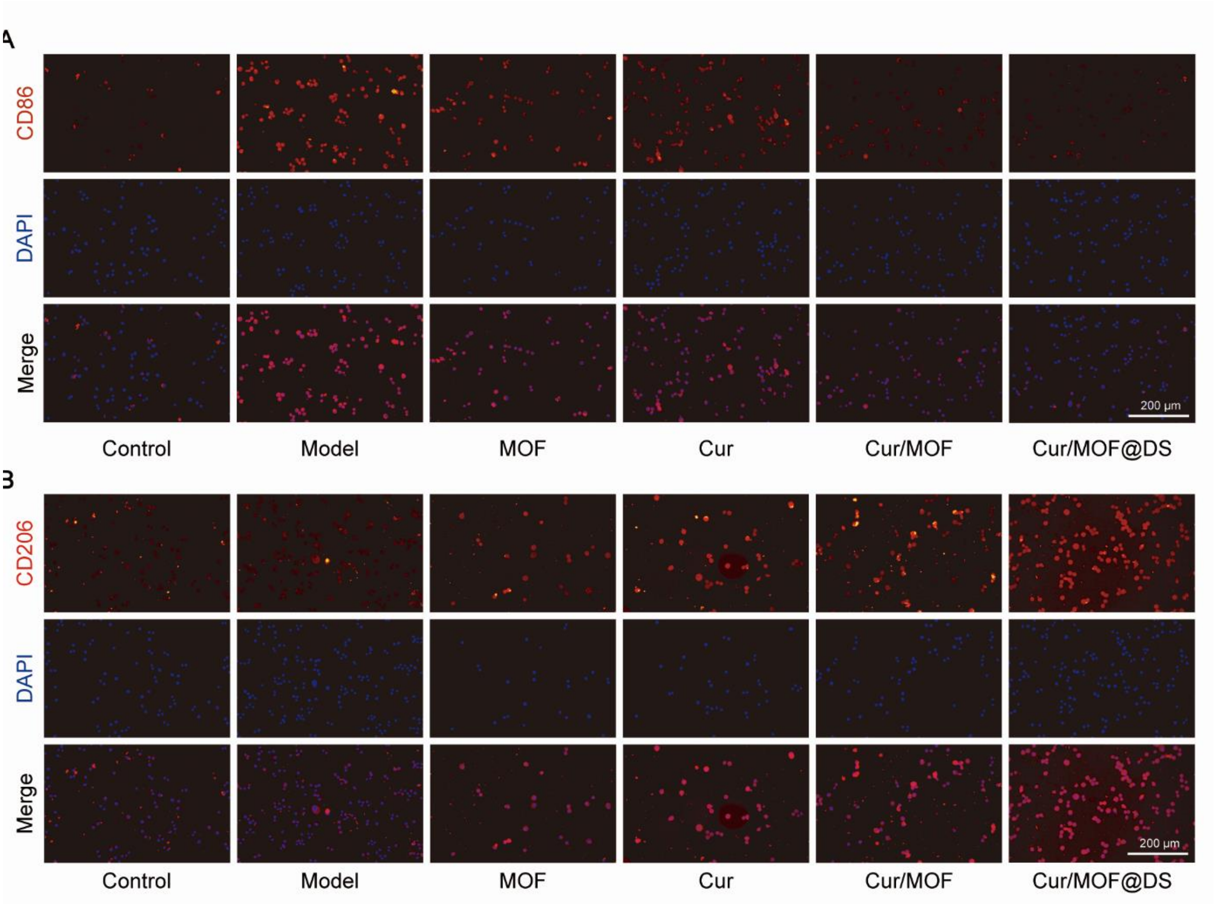

Figure S9. A, B) Fluorescence staining of Raw264.7 cells after different treatments using CD86 and CD206 antibodies. The red color represented Cy3-labeled secondary antibody, and the blue color represented DAPI-stained nucleus.

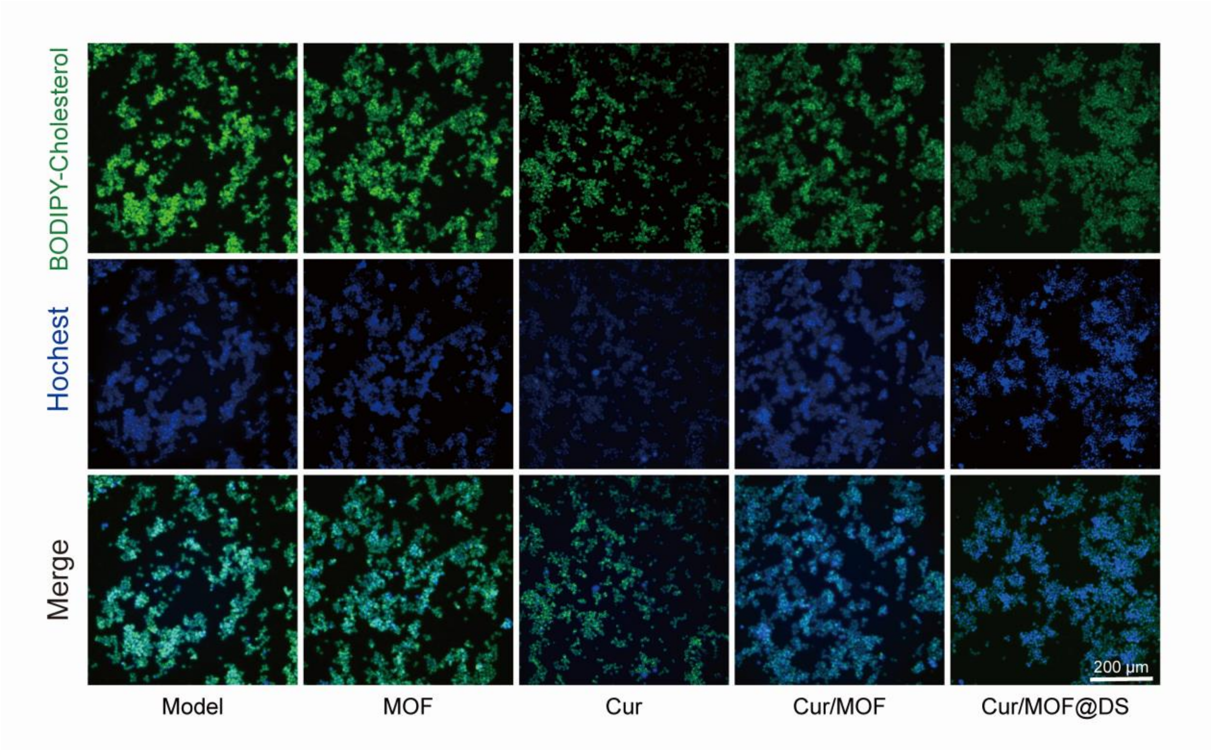

Figure S10. BODIPY-cholesterol fluorescence intensity in cells after different treatments, following overnight co-incubation of BODIPY-cholesterol with Raw264.7 cells. Blue fluorescence represents Hoechst-stained nuclei, and green fluorescence represents BODIPY-cholesterol.

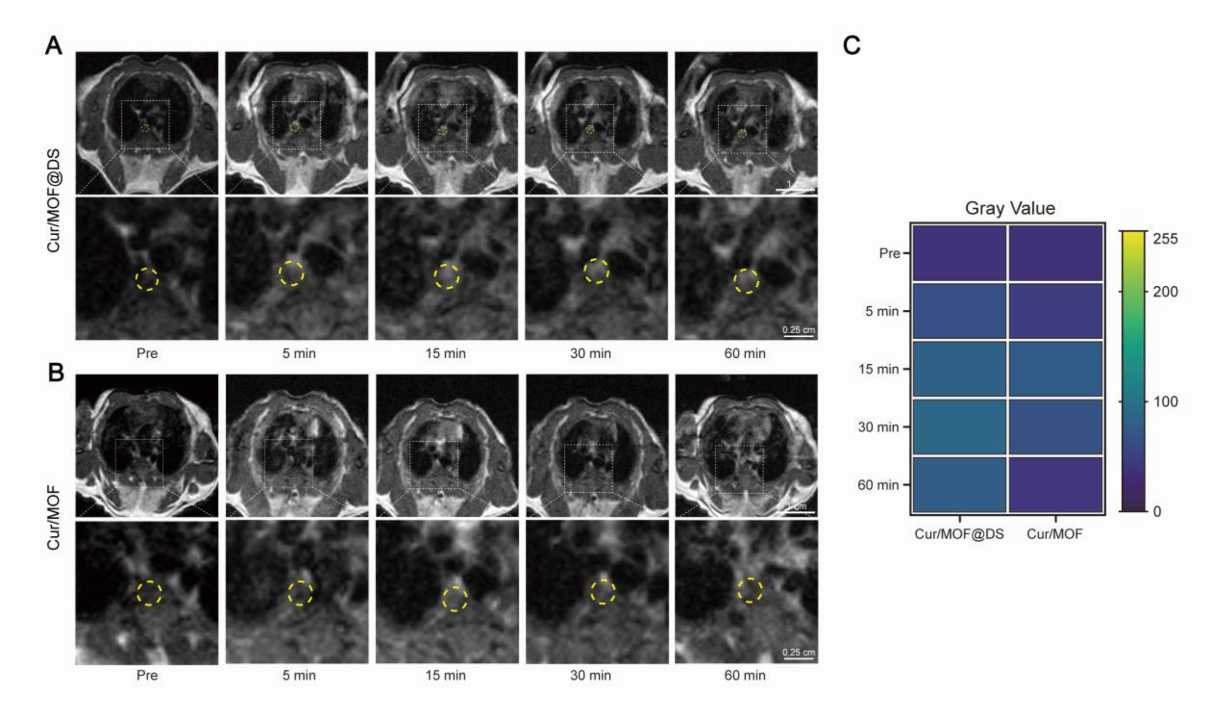

Figure S11. A, B) T1-weighted MRI of the aorta before and after tail vein injection of Cur/MOF and Cur/MOF@DS (5, 15, 30 and 60 min). The areas shown in the circle were contrast-enhanced regions of AS plaque on MRI. C) Quantitative analysis of the gray values in the contrast-enhanced regions of plaque MRI.

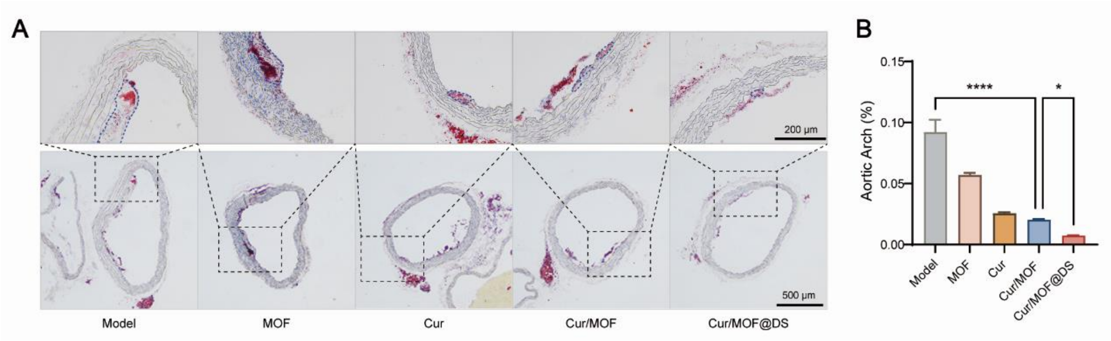

Figure S12. ORO staining and quantitative analysis of aortic arch sections after different treatments. Data are presented as mean ± SD (n = 5). *P < 0.05, ****P < 0.0001.

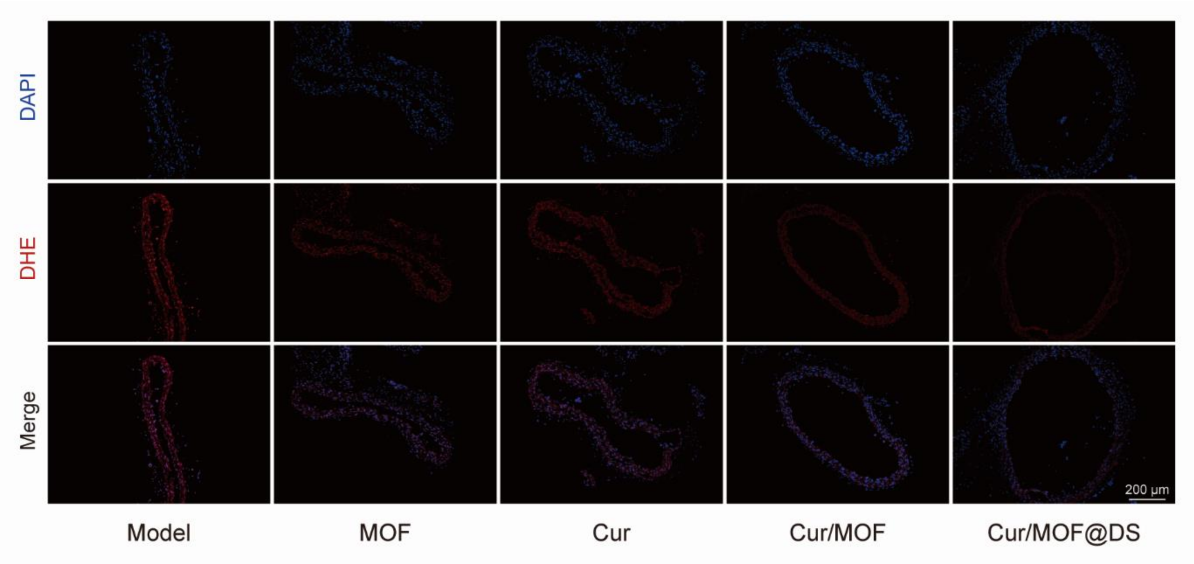

Figure S13. ROS fluorescence staining of aortic tissue frozen section in different groups. Red fluorescence represents ROS labeled with dihydroethidium (DHE), while blue fluorescence corresponds to nuclei labeled with DAPI.

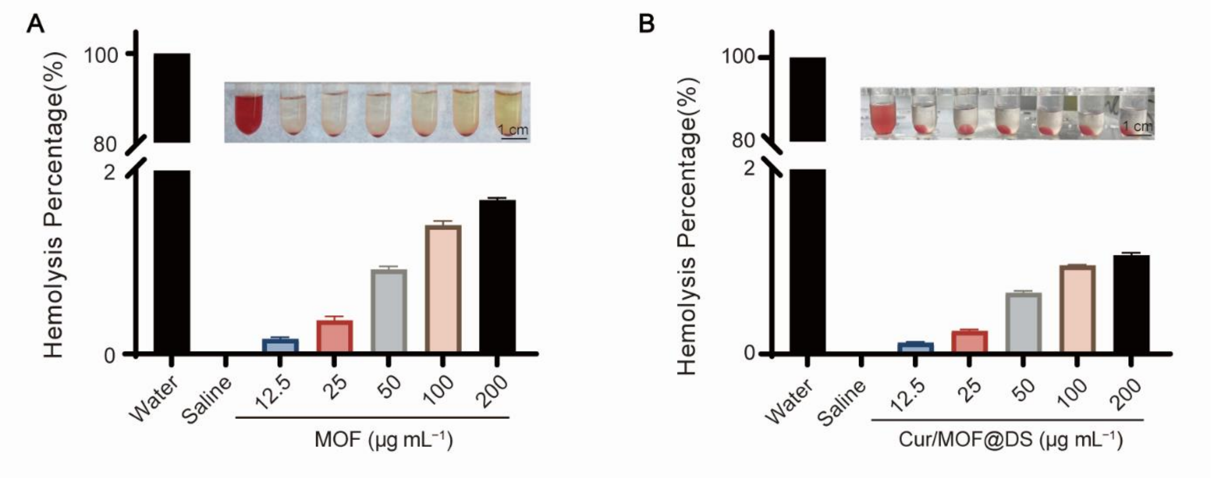

Figure S14. Hemolysis percentage of MOF and Cur/MOF@DS at different concentrations (12, 25, 50, 100 and 200 μg mL^{-1}) after co-incubation with RBC. Data are presented as means ±SD (n = 3).

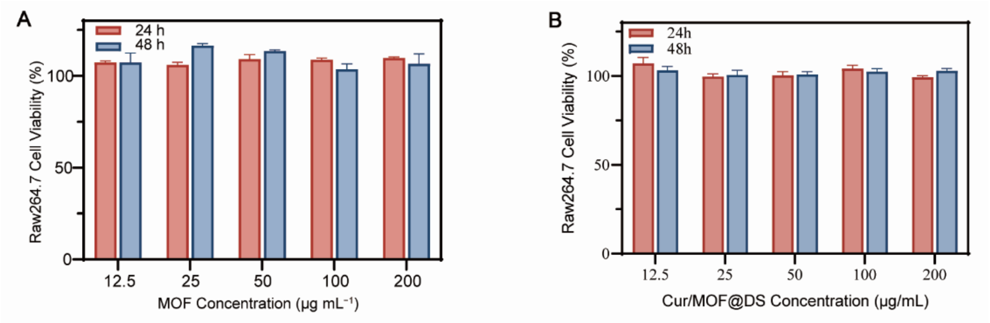

Figure S15. Cell viability of Raw264.7 cells after treatment with different concentrations of MOF (A)and Cur/MOF@DS (B) for 24 h and 48 h. Data are presented as means ±SD (n = 3).

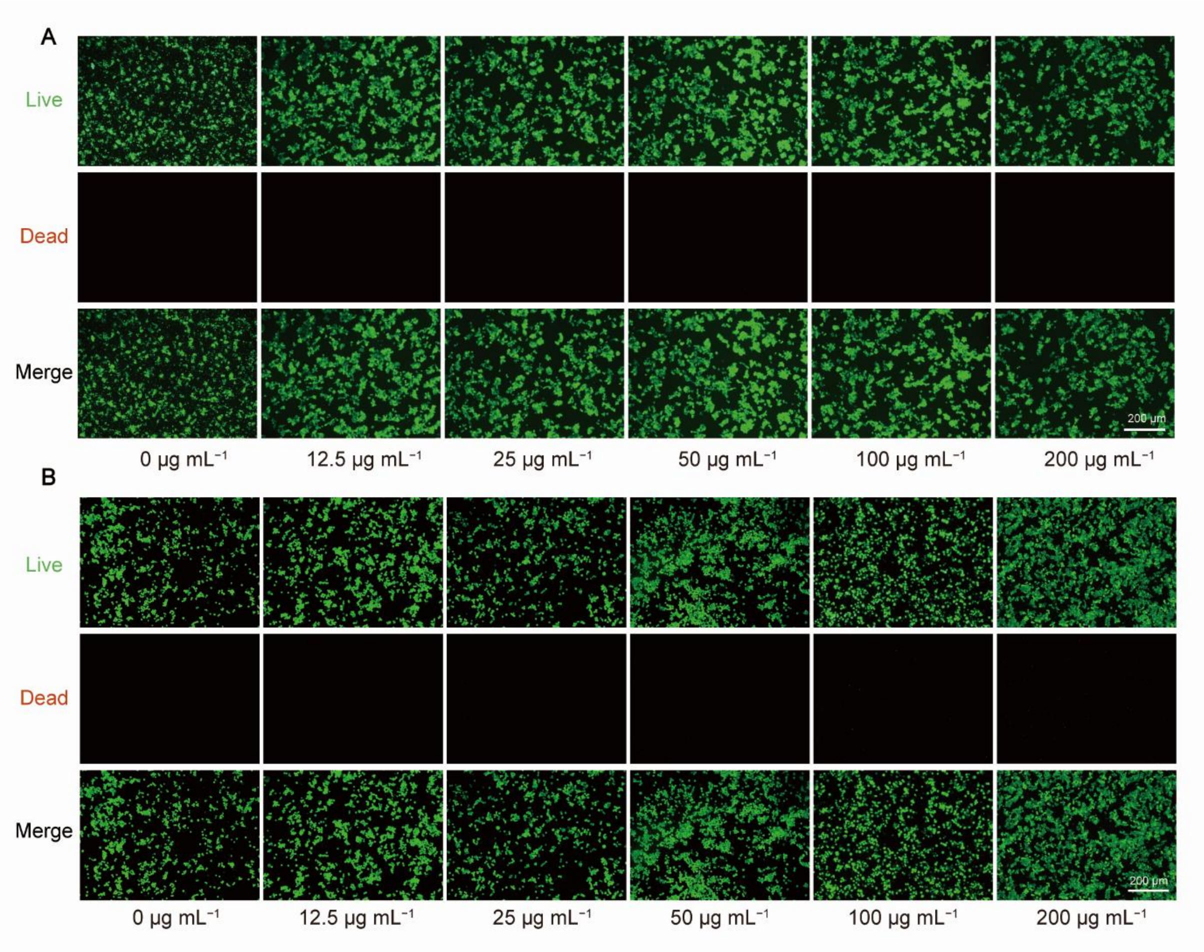

Figure S16. Fluorescence images of Raw264.7 cells after co-culture with MOF (A) and Cur/MOF@DS (B) at different concentrations for 24 h.

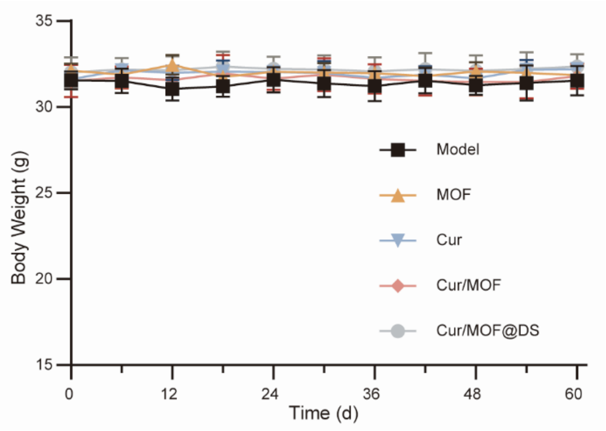

Figure S17. Changes of body weight during 8 weeks treatment. Data are presented as means ± SD (n = 5).

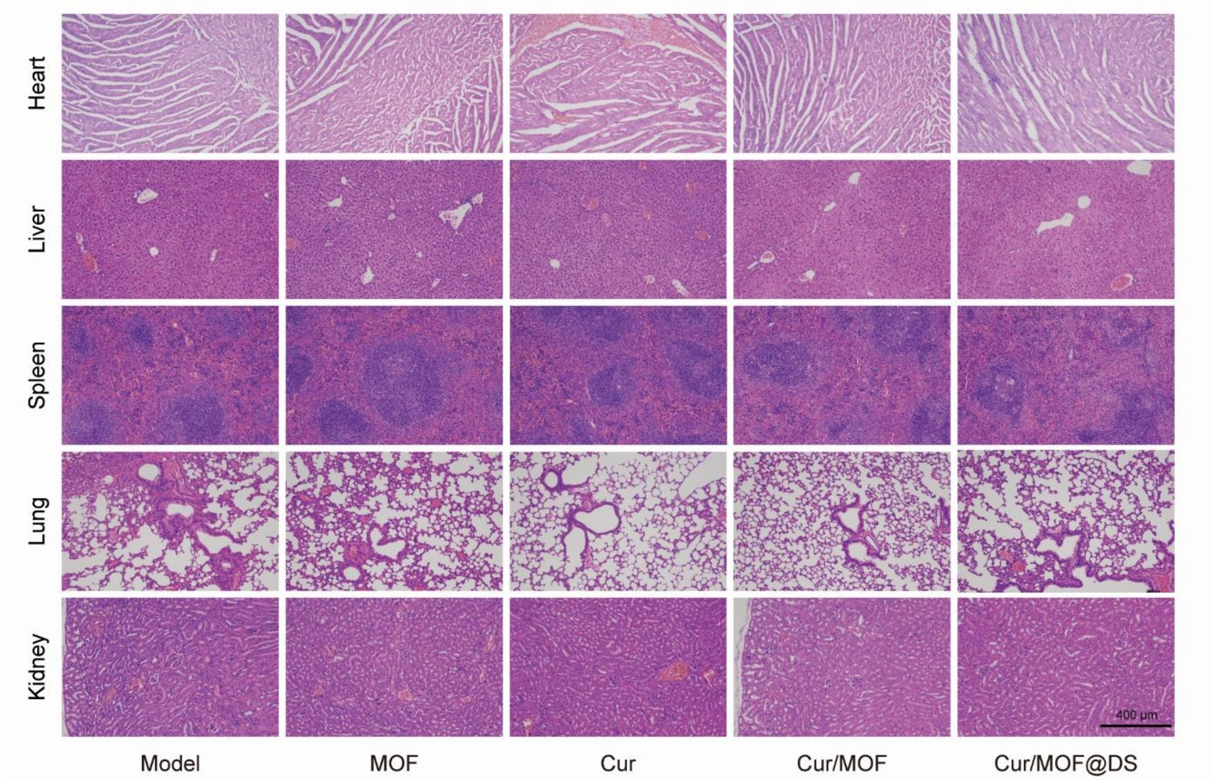

Figure S18. H&E staining images of major organs after different treatments (heart, liver, spleen, lung and kidney).

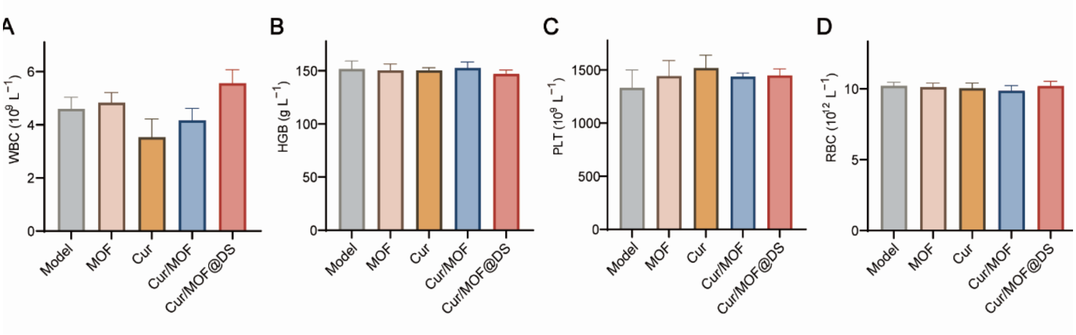

Figure S19. Levels of serum white blood cells (WBC), hemoglobin (HGB), platelets (PLT) and RBC after different treatments. Data are presented as means ± SD (n = 5).

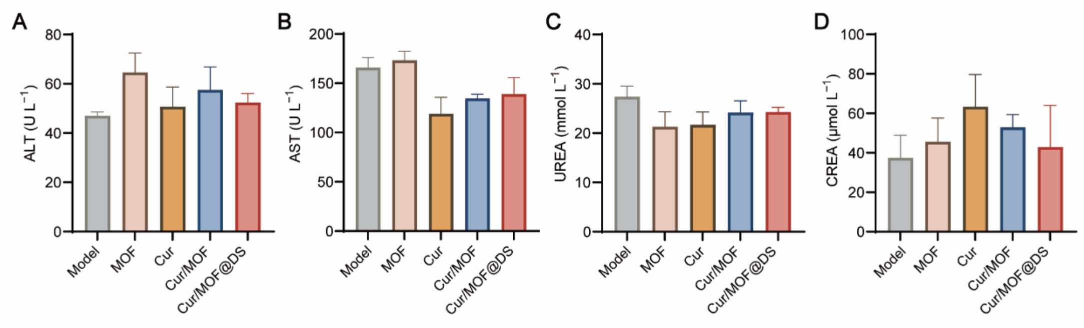

Figure S20. Serum alanine transaminase (ALT), aspartate transaminase (AST), urea nitrogen (UREA) and creatinine (CREA) levels after different treatments. Data are presented as means ± SD (n = 5).

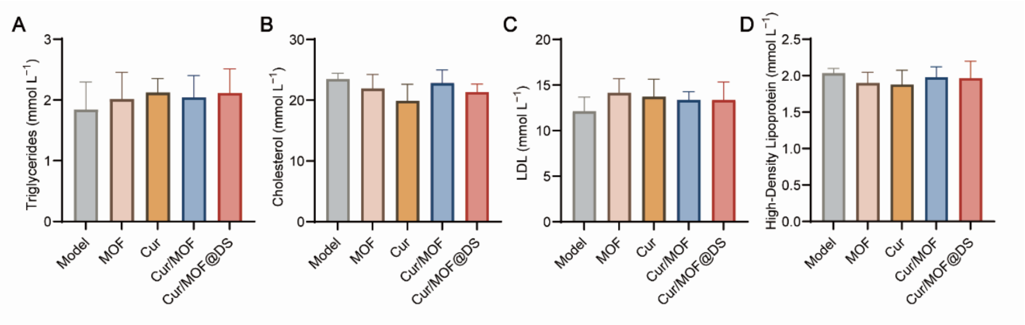

Figure S21. Blood serum lipid levels after different treatments. Data are presented as means ± SD (n = 5).
